# Comprehensive Biothreat Cluster Identification by PCR/Electrospray-Ionization Mass Spectrometry

**DOI:** 10.1371/journal.pone.0036528

**Published:** 2012-06-29

**Authors:** Rangarajan Sampath, Niveen Mulholland, Lawrence B. Blyn, Christian Massire, Chris A. Whitehouse, Nicole Waybright, Courtney Harter, Joseph Bogan, Mary Sue Miranda, David Smith, Carson Baldwin, Mark Wolcott, David Norwood, Rachael Kreft, Mark Frinder, Robert Lovari, Irene Yasuda, Heather Matthews, Donna Toleno, Roberta Housley, David Duncan, Feng Li, Robin Warren, Mark W. Eshoo, Thomas A. Hall, Steven A. Hofstadler, David J. Ecker

**Affiliations:** 1 Ibis Biosciences, Abbott, Carlsbad, California, United States of America; 2 MRIGlobal, Rockville, Maryland, United States of America; 3 United States Army Medical Research Institute of Infectious Diseases, Fort Detrick, Maryland, United States of America; 4 Chemical, Biological, Radiological, Nuclear, and Explosive Directorate, Lab Division, Pentagon Force Protection Agency, Washington, District of Columbia, United States of America; Naval Research Laboratory, United States of America

## Abstract

Technology for comprehensive identification of biothreats in environmental and clinical specimens is needed to protect citizens in the case of a biological attack. This is a challenge because there are dozens of bacterial and viral species that might be used in a biological attack and many have closely related near-neighbor organisms that are harmless. The biothreat agent, along with its near neighbors, can be thought of as a *biothreat cluster* or a *biocluster* for short. The ability to comprehensively detect the important biothreat clusters with resolution sufficient to distinguish the near neighbors with an extremely low false positive rate is required. A technological solution to this problem can be achieved by coupling biothreat group-specific PCR with electrospray ionization mass spectrometry (PCR/ESI-MS). The biothreat assay described here detects ten bacterial and four viral biothreat clusters on the NIAID priority pathogen and HHS/USDA select agent lists. Detection of each of the biothreat clusters was validated by analysis of a broad collection of biothreat organisms and near neighbors prepared by spiking biothreat nucleic acids into nucleic acids extracted from filtered environmental air. Analytical experiments were carried out to determine breadth of coverage, limits of detection, linearity, sensitivity, and specificity. Further, the assay breadth was demonstrated by testing a diverse collection of organisms from each biothreat cluster. The biothreat assay as configured was able to detect all the target organism clusters and did not misidentify any of the near-neighbor organisms as threats. Coupling biothreat cluster-specific PCR to electrospray ionization mass spectrometry simultaneously provides the breadth of coverage, discrimination of near neighbors, and an extremely low false positive rate due to the requirement that an amplicon with a precise base composition of a biothreat agent be detected by mass spectrometry.

## Introduction

Technology for detecting biothreat agents requires accurate identification of a broad array of bacterial and viral organisms that can cause severe disease and/or death, whether they occur as a result of a biological attack or from a natural source in the environment. The National Institute of Allergy and Infectious Diseases (NIAID) has compiled a list of priority pathogens for biodefense (http://www.niaid.nih.gov) and several of these are also defined as select agents (http://www.selectagents.gov/) by various agencies such as Health and Human Services (HHS) and the United States Department of Agriculture (USDA) (some of the vaccine and live attenuated strains are, however, excluded from the select agents list: http://www.selectagents.gov/Select%20Agents%20and%20Toxins%20Exclusions.html). These bioagents are often virtually indistinguishable from a group of phylogenetically related species or subspecies often referred to as “near neighbors” [1]. Near neighbors to biothreat agents may be human pathogens or harmless environmental organisms. The biothreat agent along with its near neighbors can be thought of as a *biothreat cluster or biocluster* for short. When monitoring for biothreat agents, it is important to determine whether any organisms from the biothreat clusters are present and to precisely identify the organism as a biothreat agent or a near neighbor. In some cases, the near neighbors are commonly found in the environment, and it is possible that a pathogenic near neighbor of a biothreat agent might deliberately be chosen for use in a biological attack. Thus, effective biosensor technology must be capable of identifying a broad array of biothreat agents *and* distinguishing these threats from their near neighbors unambiguously.

This requirement presents a problem for conventional molecular methods where specific PCR is used in conjunction with probes to detect specific bioagents. Not only are potentially pathogenic near neighbors present in a specimen often not distinguished, but the near neighbors sometimes react to produce false positives for the biothreat agent. To overcome these limitations, we have developed a new strategy for biothreat identification that couples biothreat cluster-specific PCR amplification to electrospray ionization/mass spectrometry (PCR/ESI-MS) [2]–[4]. The biothreat assay is performed on a hardware platform with prototypes known as TIGER [4] and as the Ibis T5000 [2], [5] that is now marketed commercially as the Abbott PLEX-ID [6]. In the PCR/ESI-MS approach, PCR primers are designed to amplify regions of the genomes of all species from the *entire* biothreat cluster, encompassing groups of organisms that include the biothreat *and* the associated near-neighbor organisms. The primers are designed to target genomic regions sufficiently conserved such that amplification occurs comprehensively within a biothreat cluster, but not outside of the cluster. The amplification products are then analyzed by mass spectrometry, which weighs the amplicons with sufficient mass accuracy that the base composition of A, G, C, and T nucleotides that make up the amplicon can be accurately counted. The base composition serves as a signature of each organism and enables identification and discrimination of the biothreat agents *and* their near neighbors with equal facility. In addition, previously undiscovered or newly emerging organisms from within these biothreat clusters are also detected. The database of signatures against which multiple additional pathogens could be identified increases over time as newer strain variants are archived and tested. An example of this was the discovery of the 2009 H1N1 virus by the Naval Health Research Center [7], [8]; this offered the first characterization of a previously unrecognized influenza strain, demonstrating the capability of the PLEX-ID in identification of a real-world case of novel pathogen emergence. Because the mass spectrometer weighs all amplicons presented to it, the amplicons from unexpected or new organisms are detected and identified [2], [5], [9], [10].

**Figure 1:**
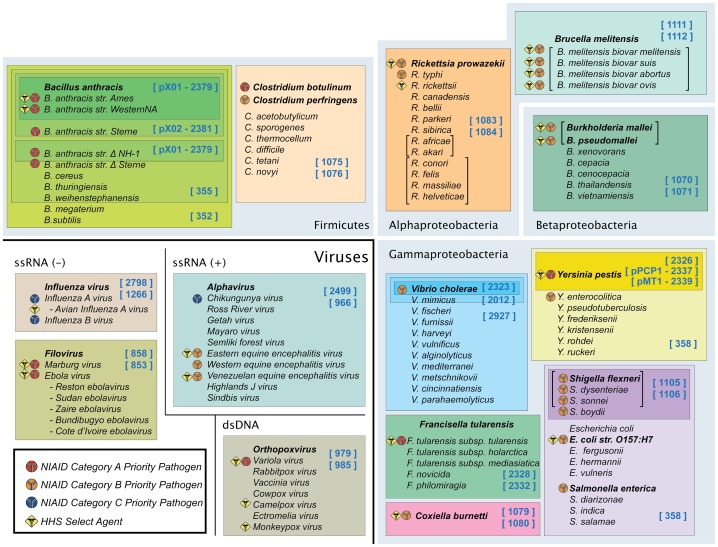
Biothreat clusters of interest. Ten bacterial and four viral clusters identified in the biothreat assay are shown. In each cluster the key biothreat agent and its near neighbors are indicated. The HHS/USDA select agent and NIAID A, B, C pathogen lists are reflected by symbols shown in the legend. Attenuated or live vaccine strains of some of these organisms are, however, excluded from the select agent list (http://www.selectagents.gov/Select Agents and Toxins Exclusions.html). *B. anthracis* and *Y. pestis* plasmid markers are explicitly annotated. Organisms with names given within brackets cannot be distinguished from each other within this assay. Primer pairs used for the detection of each biocluster are indicated.

Using this strategy, we designed a comprehensive assay to detect ten bacterial and four viral biothreat clusters. The assay identified the major biothreat organisms and differentiated these from their near neighbors and from thousands of other bacteria and viruses, providing a seamless net of biosurveillance for these clusters in a comprehensive biothreat assay (Figure 1). In this manuscript, we provide a detailed description of the methodology and the results of formal validation experiments with a variety of biothreats, near neighbors, and specimen types. We also describe several examples of how the assay has been used in real-world biothreat scenarios.

## Results

### A Comprehensive Biothreat Assay

Biothreat clusters targeted by this assay are shown in Figure 1. These organisms make up the majority of the NIAID Category A, B, and C priority pathogens and HHS/USDA select agents (http://www.niaid.nih.gov/topics/BiodefenseRelated/Biodefense/research/Pages/CatA.aspx). As shown in Figure 1, these biothreat organisms are phylogenetically related to a number of other ubiquitous organisms, making correct identification of these organisms a challenge. In addition to detecting the threat organisms, the biothreat assay described here also detects virulence factors associated with three of the agents: *Bacillus anthracis* (pXO1 and pXO2), *Yersinia pestis* (*pla* and *caf*), and *Vibrio cholera* (*ctx1*). PCR primers were designed to conserved regions within the selected target genes such that the targeted threat agent was clearly identified and differentiated from its near-neighbor species (Table 1). Many of the primer pairs used in this assay have previously been used in other assays on the biosensor system described here [2], [3], [11]–[16]. A complete analysis of each biocluster and the resolution provided by the assay is described below.

**Table 1:** Biothreat cluster detection primer pair and target sites.

BW Threat Target	Primer Pair	Gene Name	Forward Primer (5′ –>3′)	Reverse Primer (5′–>3′)
*Bacillus anthracis*	BCT352	Initiation factor IF-2	TTGCTCGTGGTGCACAAGTAACGGATATTA	TTGCTGCTTTCGCATGGTTAATTGCTTCAA
*Bacillus anthracis*	BCT355	endospore cytoplasmic protein	TCAAGCAAACGCACAATCAGAAGC	TTGCACGTCTGTTTCAGTTGCAAATTC
*Bacillus anthracis, pXO1*	BCT2381	pXO1, reverse transcriptase	TACACAGTACTGATGGTTTTGATTTCTTAGGCT	TTAGCTTTTTTGACACTTTGGTTGGATGGT
*Bacillus anthracis, pXO2*	BCT2379	pXO2, no gene name	TGGAAGTGTAAAATGTAAAAAATGGAGTCCG	TCGATTAAAGAATATGGAGATTCTTCAACGCA
*Brucella melitensis serovar abortus, melitensis, ovis, suis*	BCT1111	Ribonulcease P	TAAACCCCATCGGGAGCAAGACCGAATA	TGCCTCGCGCAACCTACCCG
*Brucella melitensis serovar abortus, melitensis, ovis, suis*	BCT1112	Ribonulcease P	TACCCCAGGGAAAGTGCCACAGA	TCTCTTACCCCACCCTTTCACCCTTAC
*Burkholderia mallei, pseudomallei*	BCT1070	Ribonulcease P	TGCGGGTAGGGAGCTTGAGC	TCCGATAAGCCGGATTCTGTGC
*Burkholderia mallei, pseudomallei*	BCT1071	Ribonulcease P	TCCTAGAGGAATGGCTGCCACG	TGCCGATAAGCCGGATTCTGTGC
*Clostridium botulinum, perfringens*	BCT1075	Ribonulcease P	TAAGGATAGTGCAACAGAGATATACCGCC	TGCTCTTACCTCACCGTTCCACCCTTACC
*Clostridium botulinum, perfringens*	BCT1076	Ribonulcease P	TAAGGATAGTGCAACAGAGATATACCGCC	TTTACCTCGCCTTTCCACCCTTACC
*Coxiella burnetii*	BCT1079	Isocitrate dehydorgenase	TCGCCGTGGAAAAATCCTACGCT	TAGCCTTTTCTCCGGCGTAGATCT
*Coxiella burnetii*	BCT1080	insertion sequence IS1111A transposase	TCAGTATGTATCCACCGTAGCCAGTC	TAAACGTCCGATACCAATGGTTCGCTC
*Francisella tularensis*	BCT2328	Aspartate semi-aldehyde dehydrogenase	TGAGGGTTTTATGCTTAAAGTTGGTTTTATTGGTT	TGATTCGATCATACGAGACATTAAAACTGAG
*Francisella tularensis*	BCT2332	Galactose epimerase	TCAGCTAGACCTTTTAGGTAAAGCTAAGCT	TCTCACCTACAGCTTTAAAGCCAGCAAAATG
*Rickettsia prowazekii, typhi*	BCT1084	Ribonulcease P	TCCACCAAGAGCAAGATCAAATAGGC	TCAAGCGATCTACCCGCATTACAA
*Rickettsia prowazekii, typhi*	BCT1083	Ribonulcease P	TAAGAGCGCACCGGTAAGTTGG	TCAAGCGATCTACCCGCATTACAA
*Vibrio cholera*	BCT2323	Cholera enterotoxin subunit A	TGCCAAGAGGACAGAGTGAGTACTTTGA	TAACAAATCCCGTCTGAGTTCCTCTTGCA
*Vibrio cholera*	BCT2927	glyceraldehyde-3-phosphate dehydrogenase	TCAATGAACGACCAACAAGTGATTGATG	TCCTTTATGCAACTTGGTATCAACAGGAAT
*Vibrio cholera*	BCT2012	Outer membrane protein	TACGCTGACGGAATCAACCAAAGCGG	TGCTTCAGCACGGCCACCAACTTCTAG
*Yersinia pestis*	BCT2339	F1 Capsule antigen	TCCGTTATCGCCATTGCATTATTTGGAACT	TAAGAGTGATGCGGGCTGGTTCAACA
*Yersinia pestis*	BCT2337	Plasminogen activator precursor	TGACATCCGGCTCACGTTATTATGGTA	TCCGCAAAGACTTTGGCATTAGGTGTGA
*Yersinia pestis*	BCT2326	insertion sequence:IS200-like and disrupted inv	TGCTGGTAACAGAGCCTTATAGGCGCA	TGGGTTGCGTTGCAGATTATCTTTACCAA
*E. coli O157:H7, S. enterica, Shigella, Yersinia pestis*	BCT358	Valine synthetase	TCGTGGCGGCGTGGTTATCGA	TCGGTACGAACTGGATGTCGCCGTT
*Shigella flexneri*	BCT1105	invasion plasmid antigen H	TGAGGACCGTGTCGCGCTCA	TCCTTCTGATGCCTGATGGACCAGGAG
*Shigella flexneri*	BCT1106	invasion plasmid antigen H	TCCTTGACCGCCTTTCCGATAC	TTTTCCAGCCATGCAGCGAC
Variola virus	VIR985	RNA helicase NPH-II	TGGAAAGTATCTCCTCCATCACTAGGAAAACC	TCCCTCCCTCCCTATAACATTCAAAGCTTATTG
Variola virus	VIR979	DNA helicase	TGATTTCGTAGAAGTTGAACCGGGATCA	TCGCGATTTTATTATCGGTCGTTGTTAATGT
Ebola virus/Marburg virus	VIR853	RNA-dependent RNA polymerase	TA/ipdU/GG/ipdU/G/ipdU/IIIIAATGTCTTTGATTGGATGCA	TG/ipdC//ipdU/A/ipdU/AAIIITCACTGACATGCATGTAACA
Ebola virus/Marburg virus	VIR858	RNA-dependent RNA polymerase	TTCATCAGGCATCATGGCACCA	TCGGCGAGGTTGTATTTCTCTAGATCAGT
Influenza Virus	VIR2798	Polymerase PB1	TGTCCTGGAATGATGATGGGCATGTT	TCATCAGAGGATTGGAGTCCATCCC
Influenza Virus	VIR1266	Nucleoprotein	TACATCCAGATGTGCACTGAACTCAAACTCA	TCGTCAAATGCAGAGAGCACCATTCTCTCTA
VEE, WEE, EEE, Chikungunya	VIR966	methyltransferase	TCCATGCTAATGCTAGAGCGTTTTCGCA	TGGCGCACTTCCAATGTCCAGGAT
VEE, WEE, EEE, Chikungunya	VIR2499	methyltransferase	TGCCAGCIACAITGTGIGAICAIATGAC	TGACGACTATICGCTGGTTIAGCCCIAC

#### Bacillus anthracis

*Bacillus anthracis*, which causes anthrax in animals and humans, is closely related to *B. cereus* (which causes human food poisoning), *B. thuringiensis* (an insect pathogen and a biological insecticide), and *B. mycoides* (considered a harmless saprophyte). Members of the this group, known as the *Bacillus cereus* clade, are environmentally ubiquitous. Classical microbiological methods can only differentiate *B. anthracis* from other near-neighbor species when the unknown isolate is shown to cause anthrax in laboratory animal models. Classical molecular phylogenetic tools, such as the analysis of rRNA gene sequences, cannot distinguish among members of the *B. cereus* clade.

We demonstrate here that two primer pairs, BCT352 targeting the translation initiation factor IF-4 (*infB)* gene and BCT355 targeting the small acid-soluble spore protein (*sspE*) gene, provide signatures that can distinguish *B. anthracis* from all of the near-neighbor species within the *B. cereus* clade. Full virulence of *B. anthracis* requires the presence of two plasmids, pXO1 and pXO2, containing three toxin components (protective antigen, lethal factor, and edema factor) and an anti-phagocytic capsule. The primers used for the detection of these two plasmids (BCT2379 and BCT2381) were chosen to capture a previously described single-nucleotide polymorphism (SNP) [17]. This region in each of the plasmids provides two different alleles that can be used to distinguish *B. anthracis* Ames and other Ames-like strains from non-Ames strains. Thus, the positive identification of the *B. anthracis* chromosome using the *infB* and *sspE* targets, combined with detection of the virulence plasmid signatures, can be used to differentiate non-pathogenic, vaccine, fully virulent, and genetically modified strains of *B. anthracis*.

To demonstrate the resolving capabilities of these genomic signatures, we obtained a collection of 34 bacilli from the United States Army Medical Research Institute for Infectious Diseases (USAMRIID). These include fully virulent, partially virulent, and avirulent *B. anthracis* strains and an assortment of near-neighbor bacilli (Table S1). Each isolate was correctly identified by comparison of base composition signatures obtained in the biothreat assay with genomic sequence data obtained from GenBank (Table 2). The *infB* locus provided identical signatures across all *B. anthracis* strains tested, whereas the *sspE* locus provided two different allelic signatures. The majority of the *B. anthracis* strains tested had a base composition signature of “A42G23C23T21,” whereas the *B. anthracis* strains from the western North American region showed a SNP at this locus and had the signature of “A41G24C23T21.” As predicted, the Ames-like strains were different from the rest of the *B. anthracis* strains in the pXO1 and pXO2 loci. An additional 89 strains of *B. anthracis* were obtained from the Keim Genetics Lab; these strains have various phylogenetic variations (Table S2). The diverse strains in this collection were correctly identified in our assay. The A1.a clade showed the SNP pattern at the *sspE* locus previously associated with the western North American lineage (Table S3). These strains were previously reported to be distinct from other Clade A strains as they contain a 153-bp allele in the CG3 locus [17].

**Table 2:** Theoretical and experimental data for *B anthracis* cluster.^1^

STRAIN	Phenotype	Data Source	GenBank Identifier	Bacillus_INFB (BCT352)	Bacillus_SSPE (BCT355)	BA_PX01 (BCT2381)	BA_PX02 (BCT2379)
Ames	pX01+/pX02+	GenBank/Complete Genome	21392688	[34 25 21 25]	[42 23 23 21]	**[40 16 22 34]**	**[45 27 13 41]**
Ames Ancestor	pX01+/pX02+		47566322	[34 25 21 25]	[42 23 23 21]	**[40 16 22 34]**	**[45 27 13 41]**
A2012	pX01+/pX02+		20520075	[34 25 21 25]	[42 23 23 21]	**[40 16 22 34]**	**[45 27 13 41]**
A0248	pX01+/pX02+		229599883	[34 25 21 25]	[42 23 23 21]	**[40 16 22 34]**	**[45 27 13 41]**
CDC 684	pX01+/pX02+		227812678	[34 25 21 25]	[42 23 23 21]	[41 15 22 34]	[44 27 14 41]
KrugerB	pX01+/pX02+		311703252	[34 25 21 25]	[42 23 23 21]	[41 15 22 34]	[44 27 14 41]
WesternNA	pX01+/pX02+		311703298	[34 25 21 25]	**[41 24 23 21]**	[41 15 22 34]	[44 27 14 41]
Sterne	pX01+/pX02-		49183039	[34 25 21 25]	[42 23 23 21]	[41 15 22 34]	Target Absent
Ames	pX01+/pX02+	Ibis MeasuredBase Counts	N/A	[34 25 21 25]	[42 23 23 21]	**[40 16 22 34]**	**[45 27 13 41]**
New Hampshire	pX01+/pX02+		N/A	[34 25 21 25]	[42 23 23 21]	[41 15 22 34]	[44 27 14 41]
Vollum	pX01+/pX02+		N/A	[34 25 21 25]	[42 23 23 21]	[41 15 22 34]	[44 27 14 41]
Vollum 1B	pX01+/pX02+		N/A	[34 25 21 25]	[42 23 23 21]	[41 15 22 34]	[44 27 14 41]
Sterne	pX01+/pX02-		N/A	[34 25 21 25]	[42 23 23 21]	[41 15 22 34]	Target Absent
STI	pX01+/pX02-		N/A	[34 25 21 25]	[42 23 23 21]	[41 15 22 34]	Target Absent
V770-NP1R	pX01+/pX02-		N/A	[34 25 21 25]	[42 23 23 21]	[41 15 22 34]	Target Absent
ATCC4728	pX01−/pX02+		N/A	[34 25 21 25]	[42 23 23 21]	Target Absent	[44 27 14 41]
Delta NH-1	pX01−/pX02+		N/A	[34 25 21 25]	[42 23 23 21]	Target Absent	[44 27 14 41]
Delta Sterne	pX01−/pX02-		N/A	[34 25 21 25]	[42 23 23 21]	Target Absent	Target Absent

1 Bolded base counts indicate SNP variants compared to the primary signature at that locus.

2 *B. anthracis* and near-neighbor organisms were obtained from USAMRIID; details provided in Supplementary Table 1.

Representative strains of the *B. cereus* clade, including 24 *B. cereus* strains and 12 *B. thuringiensis* strains were also tested (Table S2). None of these carried the virulence plasmids pXO1 or pXO2 (data not shown). Futher, the base compositions observed for the two genomic markers showed distinct signatures compared to *B. anthracis*. The amplicon for *sspE* showed a 6-bp deletion compared to the *B. anthracis* signatures, whereas the *infB* signature was the same length but had a different base composition. Based on the combined compositions of these two primer regions, the *B. cereus/B. thuringiensis* biocluster could be divided into 29 distinct genotypes. Some of the *B. thuringiensis* species have individual clusters but most are related to *B. cereus* species. Several studies over the past two decades have looked at the fine structure of the *B. cereus* clade and have reported similar findings [18]. Nucleic acids from species outside the *B. cereus* clade, such as *B. subtilis* and *B. megaterium*, were not amplified with the *sspE* primer pair. The advantage of measuring multiple signatures across the genome and in associated plasmids is that this provides sufficient information to characterize the biothreat agent (i.e., vaccine vs. virulent strains of *B. anthracis*) as well as distinguish it from its near-neighbors.

#### Yersinia pestis

The bubonic plague caused by *Yersinia pestis* is a highly contagious disease that persists endemically in many countries in the world with unpredictable resurgences [19]. *Y. pestis* is classified as a biothreat agent. It is a nonmotile, capsulated, Gram-negative bacterium transmitted to humans and susceptible animals through flea bites or aerosols. Other species in this biocluster include *Y. enterocolitica* (a diarrheagenic pathogen), *Y. frederiksenii*, *Y. ruckeri*, and *Y. pseudotuberculosis* (an enteric pathogen). *Y. pseudotuberculosis* exhibits more than 90% genomic homology with *Y. pestis,* making much of the *Y. pestis* genome unsuitable for amplification as base composition signatures do not resolve *Y. pestis* and *Y. pseudotuberculosis* [20], [21]. Two specific markers within the *Y. pestis* genome were identified that provide differentiation from *Y. pseudotuberculosis*. One of these is located in the valyl-tRNA synthetase gene (*valS*). This region (Table 1) has a SNP that is retained in all the *Y. pestis* genomes studied to date and that is distinct from the *Y. pseudotuberculosis* signature for this region (Table S4). A second primer pair was designed to target the invasin gene *invA,* a surface-expressed protein that is reponsible for cellular penetration and invasion. Although both *Y. pseudotuberculosis* and *Y. enterocolitica* contain intact invasin genes, the central region of the *Y. pestis inv* gene is disrupted by a 708-bp IS200-like element [22]. Primers targeting this region allow for the unambiguous detection of *Y. pestis* (Table S4). In addition, *Y. pestis* harbors plasmids that are required for the expression of virulence [23]–[26]. Primer pairs targeted against the *pla* gene from the *pPCP1* plasmid of *Y. pestis* and the *caf1* gene from the *pMT1* plasmid of *Y. pestis* were included in the assay to provide specific detection of virulence plasmid-carrying *Y. pestis*.

Genomic data from the completely sequenced *Y. pestis* genomes were used to verify the expected signatures for these primer pairs (Table S4). All *Y. pestis* strains for which relevant sequence was available in GenBank showed identical base composition signatures. Partially virulent *Y. pestis* strains such as *Y. pestis* Angola (PLA+/CAF−) and pestoides F (PLA−/CAF+) were correctly identified. These results were further confirmed by experimental testing a collection of seven *Y. pestis* strains with known phenotypes obtained from the USAMRIID culture collection (Table S5). In addition to the four virulent phenotypes containing PLA+/CAF+, two strains (Nairobi and Java 9) lacking the *caf* gene and pestoides F lacking the *pla* gene were analyzed. All of the results showed data consistent with the expected molecular signatures. Finally, a set of 15 near neighbors of *Y. pestis* were tested using the assay; each was correctly identified and differentiated from the *Y. pestis* signatures (Table S6). In particular, this assay clearly distinguished the *Y. pestis* signatures from those of *Y. pseudotuberculosis*, which is often the confounder in molecular assays.

#### Francisella tularensis

The *Francisella tularensis* biothreat cluster is comprised of the select agent *F. tularensis* and the near-neighbor species *F. philomiragia and F. novicida*. *F. tularensis* is the causative agent of tularemia, a disease that affects humans and other mammals; the natural reservoir is thought to be lagamorphs and rodents with ticks as the primary vector [27], [28]. *F. tularensis* has been divided into three subspecies: *F. tularensis* subsp. *tularensis* (type A), which is divided into subtypes A.I and A.II., is the most virulent and is found primarily in North America and Europe [29]. *F. tularensis* subsp. *holarctica* (type B) is less virulent and found in Europe and in Asia [28], *F. tularensis* subsp. *mediaasiatica* has been isolated only in Central Asia and is considered to be of lower virulence [30]. The latter two subspecies can cause an incapacitating infection, however [30]. The near-neighbor species *F. novicida* (considered by some investigators to be another subspecies of *F. tularensis*) and *F. philomiragia* are reportedly of of low pathogenicity and cause disease only in immunocompromised humans [30]. Because of the potential of each of these species and subspecies to cause disease of varying severity, it is important to both detect and distinguish these species and subspecies.

In the biothreat assay, the *Francisella* biocluster is identified by two genus-specific primer pairs targeting the *asd* (BCT2328) and *galE* (BCT2332) genes (Table 1). Use of *galE* for identification of *Francisella* genus has been previously described [31]. Based on inspection of the sequence alignments for these genes of all available *Francisella* sequences, both of these primer pairs are expected to generate amplicons from all known members of the *Francisella* genus. Base composition analysis of the expected amplicons shows that *Francisella* species and subspecies are distinguishable from each other (Table S7). *F. tularensis* base counts are characterized by a homogenous signature for all type A.I strains. Schu S4 is the type strain, and the rest of the *Francisella* signatures are defined here as variations compared to this reference strain. *F. tularensis* subsp. *tularensis str.* WY96-3128 (type A.II) had a T to C SNP in the *galE* primer pair region. The same signature was observed for *F. tularensis* subsp. *mediaasiatica* FSC217 strain. This is in agreement with genome-wide SNP analysis, which indicates that differentiation of these particular strains likely predated the acquisition of the *asd* or *galE* mutations that characterize the subspecies. Further, the subspecies *mediaasiatica* is reported to be closer to the Type A.II lineage, even though its pathogenicity is characteristic of the Type B strains [29]. *F. tularensis* subspecies *novicida* was distinguished by a G to A mutation from the consensus seen in *F. tularensis* signature from primer pair BCT2332.

To demonstrate the specificity of the assay for the *Francisella* biocluster, we tested a collection of 57 reference isolates obtained from the USAMRIID (Table S7). Included were members of all phylogenetic lineages within the *Francisella* biocluster. *Francisella* species were correctly identified and grouped into phylogenetic clades. All 34 Type A.I subspecies yielded identical base compositions for both primer pairs consistent with the predicted amplicons from the Schu S4 genome. Twelve of the type B, subspecies *holarctica* strains had signatures that differed from the Type A.I subspecies in both the primer pairs, clearly differentiating the two major groups of the *Francisella* genus. Type A.II strain signatures were different from the Type A.I signatures by a single T to C SNP in the *galE* primer amplicon, consistent with the genome sequence of *Francisella* subsp. tularensis strain WY96-3128. However, two of the Type B strains tested, FRAN041 (Strain JAP-Cincinnati) and FRAN011 (Strain LR) could not be distinguished from the Type A.II subspecies using these two primer pairs. As described above, this signature appears to be consistent with subsp. *mediaasiatica* strains as well. Sequence similarities between the Type B Japanese strains, *F. tularensis mediaasiatica* and Type A.II lineages were previously noted ([29], Duncan, manuscript in preparation). The *F. novicida* strain (FRAN003) differed from Type A.I by a single SNP in the *galE* region, whereas the species outlier, *F. philomiragia*, was clearly different from the rest of the *F. tularensis* biocluster in both primer regions. The *asd* primer pair produced the expected amplicon for all strains of *F. philomiragia*. In contrast, the *galE* locus primer pair did not yield an amplicon for all strains. This region in a recently sequenced *F. philomiragia* strain (ATCC 25017) has mismatches to the primer regions (Genbank Accession Number NC_010336). Even without data from the *asd* primer pair, the differentiation of this species from other *F. tularensis* was unambiguous. Based on the two primer analysis done here, *Francisella* strains could be categorized into five groups: Types “A.I,” “B,” “*novicida*,” “*philomiragia*,” and a fifth group that contains strains within the Type A.II/*mediaasiatica*/*holarctica*/Japanese lineages that might have diverged before the acquisition of the *asd* and *galE* mutations.

#### Vibrio cholerae

The genus *Vibrio*, within the family Vibrionaceae, represents a diverse group of Gram-negative bacteria that contain at least 65 described species, most of which are found exclusively in aquatic environments. Of these, at least 12 species are known human pathogens, and several other species are known to be pathogenic to marine mammals and fish. Members of this genus include *Vibrio cholerae*, the causative agent of cholera. Although large outbreaks of cholera are caused by toxigenic strains of the serogroups O1 and O139, non-toxigenic strains cause sporadic cases of disease. Other important pathogens in this group include *V. parahaemolyticus* and *V. vulnificus*, which cause significant morbidity worldwide [32].

We previously described a high-throughput pan-*Vibrio* assay for simultaneous identification of all known pathogenic *Vibrio* species [14]. The assay included broad-range PCR primers that targeted conserved sites in several housekeeping genes and the *V. cholerae*-specific toxin genes *ctxA* and *ctxB*. Base compositions from these regions were able to distinguish the various species tested and provided sub-species differentiation within the *V. cholerae* isolates. For the biothreat assay, three of the primer pairs from the pan-*Vibrio* assay were used (Table 1). One of these primer pairs was exclusive to *V. cholerae* species detection (*ompU*). One amplifies the toxin gene *ctxA*. The third primer pair, targeted to *gapA* gene, was designed to amplify most of the known species in this family and the resulting base compositions provide species-level resolution (Table S8). Genomic data analysis and experimental analysis of 42 well-characterized strains representing the phylogeny of this biocluster was used to demonstrate assay specificity.

To demonstrate the ability to detect and identify *Vibrio* spp. from natural aquatic samples, a subset of samples collected in 2006 from freshwater lakes and sites along the Georgian coast of the Black Sea were tested [14]. Six different *Vibrio* species were detected and identified in 13 of the 19 natural water samples collected from both freshwater and seawater sites spanning the seasons summer to winter in this study [14]. More than one *Vibrio* species were also detected in some samples [14]. These detections were confirmed by 16S rRNA clone library analysis.

#### Burkholderia mallei/pseudomallei

The *Burkholderia* biocluster is identified in the biothreat assay by two primer pairs, BCT1070 and BCT1071 (Table 1). The primer sets amplify conserved regions of the RNA component of ribonuclease P (RNAse P) and regions CR-IV and CR-V that bracket the highly variable extension P19. These primer pairs have homology to this gene from other proteobacteria; however, the length and base composition of the resulting amplicons are highly discriminating. The information content within amplicons from BCT1070 and BCT1071 is basically the same, and this feature is meant as a built-in redundancy check for speciation calls. Some *B. pseudomallei* strains provide the same signature as *B. mallei* (Table S9), but the next closest relative, *B. thailandensis,* is clearly segregated. Analysis of known sequences indicates that the assay will resolve most known *Burkholderia* species (with members of the *B. cepacia* biocluster being further distinguished through their distinct amplicon lengths. A notable exception is the polychlorinated biphenyl reducer *B. xenovorans*, which shows the same mass signature as *B. pseudomallei* str. 668. While this is a source of potential false positive reporting of *B. pseudomallei*, the occurrence of *B. xenovorans, which* occupies a distinct ecological niche and is not pathogenic, is quite rare.

#### Brucella

Several extremely dangerous pathogens that can infect humans and animals are found in the *Brucella* biocluster. These bacteria are easily transmitted by ingestion of unsterilized milk or meat from infected animals or close contact with their secretions or by inhalation of aerosols. *Brucella* species have slightly different preferred host specificities: *B. melitensis* infects goats and sheep, *B. abortus* infects cattle, *B. suis* infects pigs, *B. ovis* infects sheep, *B. canis* infects dogs, and *B. neotomae* infects wood rats. Taxonomists have alternated between individual species naming and naming as a single species *B. melitensis*, containing *B. melitensis* 16M and five other biovars: *abortus, canis, neotomae, ovis*, and *suis*. Recently, four additional species of *Brucella* have been described, including two that infect marine mammals. The current convention adopted by the International Committee on Systematics of Prokaryotes, subcommittee on *Brucella* (http://www.the-icsp.org/subcoms/Brucella.htm) recommends re-approval of the classical *Brucella* species with their recognized biovars.

In the biothreat assay, members of the *Brucella* biocluster are identified by two primer pairs, BCT1111 and BCT1112, that amplify two non-overlapping regions of the RNA component of ribonuclease P gene (Table 1). These two regions were chosen for their ability to amplify all *Brucella* species and to distinguish these from other near-neighbor Alphaproteobacteria species. Table S10 shows the base compositions expected from the two *Brucella* primer pairs used in the assay; signatures were derived from GenBank data for the sequenced *Brucella* species.

#### Clostridium botulinum/perfringens

The genus *Clostridium* consists of relatively large, Gram-positive, rod-shaped bacteria in the phylum *Firmicutes*. Most clostridia are opportunistic pathogens that are anaerobic, but spores are able to survive long periods of exposure to air. Most of the clostridia are saprophytes, but a few are pathogenic in humans, primarily *Clostridium botulinum, C. perfringens, C. difficile*, *and C. tetani.* Botulism is an acute neurological disease caused by a neurotoxin produced by *C. botulinum*. Eight *C. botulinum* neurotoxin types have been identified: types A, B, C1, C2, D, E, F, and G [33], [34]. Types A, B, E, and F cause human botulism. Types C and D cause most cases of botulism in animals. *C. perfringens* is classified into five types on the basis of its ability to produce one or more of the major lethal toxins, alpha, beta, epsilon, and iota. *C. tetani* is another clostridium that can be highly toxigenic to humans. Other clostridia can be highly invasive under certain circumstances.

In the biothreat assay, the clostridia are identified using two primer pairs, BCT1075 and BCT1076, targeting RNase P. These two primer pairs are capable of amplifying all members of the genus *Clostridium* and differentiate the major species (Table S11). The *C. botulinum* strains form three base composition clusters, differing from each other by one or more SNPs in each of the two amplified regions. Types A, B1, and F form a unique group and are distinguishable from all other clostridia. The second group consists of types A2, A3, and Ba4 have base compositions that overlap with *C. sporogenes*. The third *C. botulinum* group comprises types B and E and some strains of *C. perfringens* as well as the recently sequenced *C. ljungdahlii*. Most strains of *C. tetani* and the opportunistic clinical pathogen *C. difficile* form unique groups. Other clostridia that are rarely human pathogens are clearly differentiated from all the above, thus providing a rapid means of detection of the pathogenic clostridia.

#### Coxiella burnetii

Q fever is a zoonosis caused by *Coxiella burnetii*, an obligate Gram-negative intracellular bacterium [35]. *C. burnetii* infects various hosts, including humans, ruminants, and pets. Because of its highly infectious nature, *C. burnetii* is recognized as a biothreat agent. The bacterium can exist in a spore-like life cycle and remain viable and virulent for many months. Phylogenetically it occupies a unique niche, with very few near neighbors. The closest known organism based on genomic and 16S rRNA analysis is *Legionella pneumophila* [36].

In the biothreat assay, we use primers targeting isocitrate dehydrogenase (*icd*) and insertion sequence IS1111A transposase for unambiguous detection of *C. burnetii* (BCT1079 and BCT1080, respectively). The latter occurs in multiple copies in the bacterium (from 5 to 31 copies) [35], [37]. Base composition analysis of the expected products showed that all sequenced *Coxiella* genomes (NM, Dugway, K, and Q) share identical signatures in both amplified regions (Table S12). These primer pairs do not amplify Legionella (data not shown) and should not amplify nucleic acids from any of the other proteobacteria. The *C. burnetii* G (Q212) sequenced genome showed two different base counts at the IS111A locus, suggesting a SNP variant in this region, similar to operonic diversities often seen in 16S rRNA sequences for other bacteria.

#### Rickettsia prowazekii

The *Rickettsiaceae* are a family of obligate intracellular small Gram-negative coccobacilli that infect humans chiefly through insect vectors, mostly from animal hosts [38]. The rickettsial fevers are acute bacteremic illnesses characterized by headache, mental confusion, and, in severe cases, meningoencephalitis. The genus *Rickettsia* is divided into three main groups: *R. prowazekii*, the agent of classical epidemic typhus; *R. typhi*, the causal agent of endemic typhus; and the “spotted fever” group of rickettsiae, which contains a large number of species transmitted from rodents, dogs, and wild animals by ticks. The latter group includes *R. rickettsii*, the agent of Rocky Mountain spotted fever; *R. conorii*, the cause of tick typhus in the Mediterranean area and in India; *R. africae*, which is found in the African veld; *R. japonica*, *R. australis*, and a variety of other similar organisms that are widely distributed in Asia and Australia.

Organisms in the *Rickettsia* biocluster are identified by two primer pairs, BCT1083 and BCT1084, which prime different regions of the RNA component of the RNase P. Since the primers target *Rickettsia*-specific sequences, no amplification is expected outside the *Rickettsiaceae* family. Using these two primer pairs, members of the Rickettsia biocluster can be distinguished from each other. In particular, *R. prowazekii*, *R. typhi*, and *R. rickettsii* species yield distinct PLEX-ID base composition clusters (Table S13).

#### Enterobacteriaceae (Escherichia coli O157:H7, Shigella, Salmonella enterica)

Diarrheagenic enterobacteria, such as *E. coli*, *Salmonella*, and *Shigella* species, are closely related organisms that are ubiquitous food and water-borne pathogens. These agents have the potential to cause significant damage to the food supply and are high-risk clinical pathogens. There are over 3,500 *Salmonella* subtypes, and all are human pathogens. The majority of these serotypes belong to a single *Salmonella* species, *Salmonella enterica,* which includes six subspecies (subsp. *enterica*, subsp. *salamae*, subsp. *arizonae*, subsp. *diarizonae*, subsp. *houtenae*, and subspecies *indica*). For *Shigella*, there are four species (*Shigella dysenteriae*, *S. flexneri*, *S. boydii*, and *S. sonnei*); all can cause enteric illnesses. There are at least five pathotypes of *E. coli*: enteroinvasive *E. coli* (EIEC), enterotoxigenic *E. coli* (ETEC), enteropathogenic *E. coli* (EPEC), enterohemorrhagic *E. coli* (EHEC), and enteroaggregative *E. coli* (EAEC). Symptoms caused by these organisms are often similar, and these organisms are difficult to differentiate by genomic analysis.

In the biothreat assay, a primer pair targeting the valine synthetase (BCT358) gene provides identification of all of the above species in the *Enterobacteriaceae* family. In addition, this primer pair also provides species resolution of *Yersinia* as discussed previously. The base count clusters shown in Table S14 demonstrate the ability of this primer pair to amplify members of the family *Enterobacteriaceae* and to provide species-level differentiation of *Salmonella* from *E. coli* and *Shigella*, although the *Shigella* species base counts are indistinguishable from a group of the *E. coli* strains using this primer pair (PLEX-ID cluster 5, Table S14). This is consistent with the previously described phylogenetic relationship of these bacteria [39]. Importantly, the pathogenic *E.* *coli* O157:H7 species is clearly differentiated from all other *Enterobacteriaceae* except enteropathogenic *E. coli* O55:H7, strain 5905 (BC cluster 1). This strain is considered the immediate ancestor of the *E. coli* O157:H7 lineage and contains the shiga toxin, which is atypical for other *E. coli* O55:H7 strains [40]. The *Salmonella* species are divided into several clusters, but cannot be grouped according to the subspecies nomenclature based on data from this assay. These pathogens can be resolved in a more targeted food-borne bacteria assay (manuscript in preparation).

In order to provide additional separation between *E. coli* and *Shigella* species, we added two more primer pairs targeting two different regions of invasion plasmid antigen H (*ipaH*). As shown in Table S15, these two primer pairs amplify all *Shigella* species but do not amplify *E. coli* or *Salmonella* (data not shown). Thus, the four major *Shigella* species can be clearly identified as a group distinct from *E. coli* using information from the three primer pairs. This assay, however, does not distinguish among the various *Shigella* species.

#### Alphaviruses

The genus *Alphavirus*, of the family Togaviridae, contains at least 37 species and subtypes or varieties and at least 23 have been associated with human illness. Some New World *Alphavirus*, such as the eastern equine encephalitis viruses (EEEV) and Venezuelan equine encephalitis virus (VEEV) complex, are considered potential bioweapons [41]. Other important members of this virus group include the western equine encephalitis virus (WEEV) complex viruses that include Sindbis virus. The Old World cluster includes Chikungunya virus and Semiliki Forest virus complex among others. We have previously described an assay for pan-*Alphavirus* detection and demonstrated the utility of this with field-collected mosquito and clinical samples [12]. This assay detects a wide variety of *Alphavirus* in naturally occurring biological backgrounds and was used to identify a virus that was a novel subtype IIID in the VEEV complex [12]. Two of the three primer pairs described previously were used in the biothreat assay and have been tested against a broad panel *Alphavirus* isolates representing both the Old World and New World *Alphavirus* (Table S16). Both are targeted to the NS1 region on the 5′-end of Alphavirus genome. Primer pair VIR966 exhibited the greatest breadth of coverage. Base counts from this primer pair amplicon alone were sufficient to distinguish most of the isolates at the species and strain level. The second primer pair (VIR2499) used in the study contains inosine (I) nucleotides at selected sites in both the forward and reverse primers to enhance hybridization, but despite this, did not amplify several of the Old World *Alphavirus*. All the *Alphavirus* samples tested were detected with at least the VIR966 pair and most were identified to strain or subtype level.

#### Orthopoxvirus

The genus *Orthopoxvirus* contains several species of related viruses including the causative agent of smallpox (*Variola* virus). In addition to smallpox, several other members of the genus are capable of causing human infection, including monkeypox, cowpox, and other zoonotic rodent-borne poxviruses. We have previously described a pan*-Orthopoxvirus* assay for identification of all members of the genus based on four PCR reactions targeting *Orthopoxvirus* DNA and RNA helicase and polymerase genes. The assay can detect and identify diverse orthopoxviruses, provide sub-species information, and characterize viruses from the blood of rabbitpox virus-infected rabbits [11]. In the biothreat assay, we used two of these four primer pairs (VIR979 and VIR985). The two provide species-level resolution of the genus *Orthopoxvirus* and, in particular, differentiate the *Variola* and monkeypox viruses from each other and from vaccinia, rabbitpox, and ectromelia viruses (Table S17).

#### Influenza virus

Influenza A viruses are important respiratory pathogens that cause annual epidemics and occasional pandemics. Influenza viruses cause serious global economic and public health burdens. Emergence of new influenza A virus strains can be caused by “antigenic shift,” resulting from reassortment of gene segments (including H and/or N types), by “antigenic drift” resulting from the continuing accumulation of mutations in the H and N genes, or by species jump by a virus that acquires the ability to infect and be transmitted among humans as has happened in the influenza pandemics over the last century [42], [43]. In April 2009, a previously unseen virus emerged and rapidly spread globally leading to the first influenza pandemic of the 21^st^ century. The continuous evolution of influenza genomes together with reassortment events pose challenges to the effective monitoring of influenza viruses. We previously described an RT-PCR/ESI-MS assay for the detection and characterization of influenza viruses [8], [10], [15], [16], [44], [45]. Identification of each influenza A virus is based on the summation of base composition signatures obtained from the six to eight primer pairs.

In the biothreat assay, two of the previously described sets of primer pairs (VIR2798 and VIR1266) were included for rapid detection of presence of influenza A or B virus. Base composition data from the amplified regions of over a 1000 influenza A H5N1 strains from GenBank were analyzed. The majority of these could be grouped into the six base composition clusters as shown in Table S18. These clusters were unique and distinct from other avian and non-avian signatures (data not shown), with the exception of two instances of avian H9N2 sharing base composition overlap with one or more of the avian H5N1 clusters. In both these instances, however, all the overlapping strains were from local outbreaks (Shantou 2003 and Guangxi 2006) and were not widely distributed. Similar correlations were found for pandemic 2009 H1N1, seasonal H3N2, and seasonal H1N1 viruses as previously described [44]. In the biothreat assay, the non-avian H5N1 subtypes will be reported only at the species level as influenza A virus. Further differentiation of the sub-species may be achieved using the broader PCR/ESI-MS influenza assay previously described [13].

#### Filovirus

*Filoviridae* is a viral family of negative-strand RNA viruses that include two major genera, *Ebolavirus* and *Marburgvirus*, both of which contain highly pathogenic and potential biowarfare agents. Some of the species in these groups include Sudan, Reston, Zaire, and Cote d’Ivoire ebolavirus and several strain variants of the Lake Victoria marburgvirus species. We have developed an assay for the detection of all members of this family using two primer pairs targeting the RNA-dependent RNA polymerase region of the genome. There is significant sequence diversity among the filoviruses and in order to ensure primer hybridization to all the above viruses, we used modified nucleotides in the PCR primer pairs as previously described for the detection of the SARS coronavirus [9]. We tested these primers with samples obtained from the CDC Special Pathogens Branch (Dr. Stuart Nichol, personal communication). In all cases, we obtained the base composition signatures expected based on sequenced genomes of these viruses (Table S19). Due to the highly pathogenic nature of these viruses, these viruses were not used in any additional analytical characterization studies described below.

### Data Analysis and Reporting

The above sections describe in detail the primer pairs used in the biothreat assay and the ability of the individual groups of primer pairs to detect the targeted biothreat cluster. All of these primer pairs were assembled into a single assay kit containing groups of two to three primer pairs per well for screening for all the listed biothreat agents simultaneously. The assay layout is shown in Figure S1. Sixteen wells of a 96-well microtiter plate were utilized for analysis of each sample. Up to six samples may be screened per plate. Importantly, each PCR well included a synthetic DNA calibrant that was amplified by one of the target primer pairs. This calibrant served as a PCR positive control and allowed relative determination of the quantity of the target organism as previously described [2], [4].

Data analysis and reporting for this assay were optimized for detecting the targeted biothreat clusters, and detection of organisms outside this group are not reported. Two different types of report are currently available. The first is a summary that reports detection and lack thereof for each of the 14 groups described in the previous sections for each sample (Figure S2). The criteria for inclusion in this report are the detection of the biothreat cluster organisms in one or more primer pairs targeting the individual clusters. The primer pairs targeting the plasmid markers are reported separately. In the specific example shown, the test organism was a *B. anthracis* strain containing both virulence plasmids. The report indicates detection of the genome and the two plasmids. The approximate genome equivalents per well for the target organism are based on relative amplification compared to the calibrant. At higher target organism concentrations, the calibrant is often outcompeted in the PCR well; therefore, the reported levels at higher loads could be inaccurate. Another more in-depth analysis of the data is available that provides base composition details to support the calls reported in the summary (Figure S3).

### Analytical Performance Characteristics

#### Analytic sensitivity

The limit of detection (LOD) of the biothreat assay in analysis of environmental aerosol samples, one of its intended uses, was determined by analyzing serial 10-fold dilutions of nucleic acids from a number of test agents with air filter nucleic acid extract from a biodefense monitoring program (“*Dirty Air*”). In parallel, samples were also tested in DNA elution buffer (*TE buffer*) alone. Fivefold serial dilutions of nucleic acid samples (with a high concentration of 1000 GE/well for all targets except VEEV which was 5000 GE/well) of the nucleic acid extracts from all the target organisms were prepared and 10 replicates of each dilution of each agent were analyzed. The presumptive LOD was ascribed to be the lowest concentration resulting in 10 correct identifications and detections in all primer pairs targeting each biothreat cluster (Figure S4). Additional replicates were analyzed (as many as 105) to allow for sufficient data to determine the LOD with a 95% confidence interval. These measured LODs were used for subsequent analysis. Nucleic acids from test organisms were paired to reduce overall sample numbers (for example, *B. anthracis* and vaccinia virus nucleic acids were spiked together and analyzed in the same sample). These LOD studies were preceded by analysis of synthetic constructs, plasmids containing DNA sequences similar to those of the biothreat agents. These constructs were carefully measured using real-time PCR assays targeting the plasmid backbone (data not shown). Using this synthetic approach, the LOD for the various target primer pairs in the biothreat assay were determined to be between 7 and 250 genome equivalents (GE) per well, with most organisms detected at between 15 and 62.5 GE/well (Figure S4). The outliers were the three RNA virus groups, which showed either 125 or 250 GE/well LOD.

The LODs and the false negative rates for all threat agents tested are summarized in Table 3. The LOD for the threat agents tested in the context of environmental air background ranged from 40 to 1000 GE/well. It was found that 37.5% (6/16) of the threat agents tested had LODs of 40 GE/well, 50% (8/16) had LODs of 200 GE/well, and 12.5% (2/16) had LODs of 1000 GE/well. At the LODs, false negative rates were less than 5% for 14 of the agents used to challenge the biothreat assay kit and less than 10% for two of the agents. Detection and identification of all the threat agents relies on more than one primer pair; in the case of *B. anthracis*, it relies on four primer pairs. For calculation of LOD, all targeted primer pairs were expected to amplify and produce results. However, in routine operation, any primer pair producing a result would result in an organism identification. The false negative rates were calculated based on a failure to detect the threat organism.

**Table 3:** Limits of detection and false negative rates.

Threat	GE/well	Percent complete	Correct	False Negative Rate	UL (95% Confidence)
*Bacillus anthracis*	200	98%	104/109	4.6%	9.3%
*Brucella melitensis*	1000	100%	93/96	3.1%	7.8%
*Burkholderia mallei*	200	100%	96/97	1%	4.9%
*Clostridium botulinum*	40	100%	96/96	0%	3.3%
*Clostridium perfringens*	200	100%	94/96	3.1%	6.4%
*Coxiella burnetii*	200	100%	96/96	0%	3.3%
*E. coli* O157:H7	200	100%	89/96	7.3%	13.1%
*Francisella tularensis*	40	100%	96/96	0%	3.3%
Vaccinia virus	200	100%	96/96	0%	3.3%
*Rickettsia prowazekii*	1000	100%	96/96	0%	3.3%
*Salmonella enterica*	40	100%	95/96	1%	4.9%
*Shigella flexneri*	200	100%	96/96	0%	3.3%
VEE	200	100%	96/96	0%	3.3%
*Vibrio cholerae*	40	100%	95/97	2%	6.3%
*Y. pestis*	40	100%	95/96	1%	4.9%

#### Specificity

It is critical that an assay used for biodefense monitoring be capable of detecting threats and, perhaps as importantly, of not falsely identifying a threat. The sample preparation methods employed by environmental monitoring programs result in samples containing significant amounts of nucleic acids from a variety of species. Two methods were employed to determine the specificity of the biothreat assay. First, over 1000 samples containing environmental background without a specific target agent were analyzed and used to calculate false positive rates for each agent. Second, the ability of the biothreat assay kit to detect a threat when the sample contained both the targeted threat and a near neighbor but not when the sample contained only the near neighbor was assessed.

A total of 1,353 samples in an environmental air background were analyzed during the determination of sensitivity. Each of these samples contained only two of the agents under investigation. Because the biothreat assay simultaneously analyzes each sample for every threat agent, the results from those samples not containing a particular threat agent were used to determine the false positive rates of that agent. For example, in the LOD determination of *B. anthracis*, the sample contained nucleic acids extracted from the environmental collection and from *B. anthracis* and vaccinia virus. Because those samples did not contain any of the other 14 threat agents, the data from the analysis of those samples could be used to calculate the false positive rate. The false positive rates for each of the threat agents tested was 0%, except for *Rickettsia prowazekii,* which was 14% (Table 4). The matrix that was used as background had high loads of environmental bacterial signatures (data not shown), including alphaproteobacteria such as *Rickettsia* and *Mesorhizobium* species. These might account for the observed higher false positive rates for rickettsial species compared to other organisms. Higher background prevalence of some of the potential biothreat agents or their near neighbors in certain regions of the world might similarly affect other detections. For instance, *Burkholderia pseudomallei*, which is described as a highly distributed soil saprophyte in southeast Asia would result in higher rates of reporting of this organism.

**Table 4:** Specificity of the biothreat assay measured as false positive rates.

Threat	Detection	False Positive Rates	UL (95% Confidence)
*Bacillus anthracis*	0/1192	0%	0%
*Francisella tularensis*	0/1206	0%	0%
*Yersinia pestis*	0/1198	0%	0%
*Vaccinia Virus*	1/1198	0%	0%
*Brucella melitensis*	0/1203	0%	0%
*Vibrio cholerae*	0/1135	0%	0%
*Burkholderia mallei*	0/1205	0%	0%
*Salmonella enterica*	0/1209	0%	0%
*VEE*	0/1169	0%	0%
*E. coli O157:H7*	0/1182	0%	0%
*Influenza A virus*	0/1270	0%	0%
*Clostridium perfringens*	0/1170	0%	0%
*Clostridium botulinum*	0/1207	0%	0%
*Shigella flexneri*	0/1157	0%	0%
*Coxiella burnetii*	0/1205	0%	0%
*Rickettsia prowazekii*	167/1193	14%*	13.9%*

Near-neighbor nucleic acids were added to the sample in excess (fivefold higher than the LOD) of the target biothreat nucleic acids (added at twofold the LOD). The results are presented in Table 5. For each of the threat agents tested, the threat organism was correctly identified by the PLEX-ID system even when excess near-neighbor nucleic acids were present in the sample. As expected for the *Brucella melitensis* cluster, the PLEX-ID biothreat assay did not discriminate between *B. melitensis, B. abortus, B. suis, and B. ovis*, which are all considered to be potential threat organisms. When *Rickettsia canadensis* was included during testing of the Rickettsial primer pairs, *Coxiella burnetii* was also identified as a false positive. This detection was reported by the Coxiella primer pairs, not the rickettsial primer pairs, indicating that there was a possible contamination of the *R. canadensis* with *Coxiella burnettii*. Thus, the PLEX-ID clearly demonstrated the ability to detect threat agents in the presence of both background and excess near-neighbor nucleic acids.

**Table 5:** Specificity of the biothreat assay as measure by near-neighbor challenge.

Spiked nucleic acid extracts (Concentration)	Type: Biothreat (BT) orNear Neighbor (NN)	Organisms identified
*Bacillus anthracis* (400 GE/well)	*BT*	*Bacillus anthracis*
*Bacillus thuringiensis* (1000 GE/well)	*NN*	*Bacillus thuringiensis/cereus* 1
*Brucella melitensis* (2000 GE/well)	*BT*	*Brucella melitensis/abortus/suis/ovis* 2
*Brucella abortus (*5000 GE/well)	*BT*	
*Burkholderia mallei* (400 GE/well)	*BT*	*Burkholderia mallei/pseudomallei*
*Burkholderia cepaciae* (1000 GE/well)	*NN*	*B. cepacia/cenocepacia/Burkholderia sp. 383*
*Clostridium botulinum* (400 GE/well)	*BT*	*Clostridium botulinum/sporogenes*
*Clostridium difficile* (1000 GE/well)	*NN*	
*Clostridium perfringens* (400 GE/well)	*BT*	*Clostridium* perfringens^3^
*Clostridium difficile* (1000 GE/well)	*NN*	
*Coxiella burnetii* (400 GE/well)	*BT*	*Coxiella* burnetii^3^
*Legionella pneumophila* (1000 GE/well)	*NN*	
*Francisella tularensis* (400 GE/well)	*BT*	*Francisella tularensis* 3
*Francisella philomiragia* (1000 GE/well)	*NN*	
*Rickettsia prowazekii* (2000 GE/well)	*BT*	*Rickettsia prowazekii*
*Rickettsia canadensis (*5000 GE/well)	*NN*	*Rickettsia Canadensis*
		*Coxiella burnetii* 4
*Vibrio cholera* (80 GE/well)	*BT*	*Vibrio* cholera^3^
*Vibrio vulnificus* (200 GE/well)	*NN*	
*Y. pestis* (80 GE/well)	*BT*	*Y. pestis* 3
*Y. frederiksenii* (200 GE/well)	*NN*	
*Escherichia coli* O157:H7 (400 GE/well)	*BT*	*Escherichia coli* O157:H7/E. coli
*Klebsiella pneumoniae* (1000 GE/well)	*NN*	*Klebsiella pneumoniae/ozaenae*
*Shigella flexneri* (400 GE/well)	*BT*	*Shigella flexneri/dysenteriae/boydii*/*sonnei*
*Klebsiella pneumonia* (1000 GE/well)	*NN*	*Multiple Klebsiella sp*
		*Multiple Escherichia sp*
*Salmonella enterica* (80 GE/well)	*BT*	*Salmonella enterica/salamae* 3
*Klebsiella pneumonia* (200 GE/well)	*NN*	

1 Indicates inability to differentiate the species further.

2 *Brucella* is identified at the genus level in this assay.

3 Near-neighbor organism not detected.

4 *C. burnettii* detected by Coxiella primer pairs; potentially contaminated *R. Canadensis* stock.

#### Breadth of coverage

To determine the ability of the biothreat assay to distinguish between threat agents and near neighbors, the biothreat assay was challenged with nucleic acids purified from a panel of organisms. These samples were diluted in Tris-EDTA (pH 8.0) to allow for analysis on the biothreat assay plate at 1000 GE/well for each organism individually. In every case, the challenge organism was correctly identified. When possible, additional strains or sub-species were also identified. However, the intended use of the biothreat assay is to alarm the end user when a threat is present. The data presented in Table 6 clearly demonstrate the ability of the assay and the instrument to discriminate between threat agents and near neighbors.

**Table 6:** Organisms appropriately detected as threat or near neighbor.

CRP Cat#	Organism	Type: Biothreat (BT) or Near Neighbor (NN)
BACI002	*B. anthracis* V770-NP-1R	BT
BACI012	*B. anthracis* Sterne	NN; (pXO2-)
BACI055	*B. anthracis* Pasteur-Like	NN (pXO1-)
BACI124	*B. anthracis* Vollum 1B	BT
BACI126	*B. anthracis* Pakistan SK-102	BT
BACI123	*B. anthracis*	BT
BACI207	*B. anthracis* South Africa	BT
BACI225	*B. anthracis* RA3	BT
BACI228	*B. cereus* 3A	NN
BACI232	*B. cereus* G9241	NN
BACI020	*B. coagulans* 7050	NN
BURK003	*B. pseudomallei* 1026B	BT
BOTB	*C. botulinum* B	BT
FRAN017	*F. philomiragia* 25016	NN
FRAN003	*F. tularensis* var *novicida* 15482	NN
FRAN004	*F. tularensis LVS* var *palearctica*	NN
FRAN012	*F. tularensis*	BT
FRAN016	*F. tularensis* Schu S4	BT
FRAN029	*F. tularensis*	BT
RICK002	*R. prowazekii* Cairo	BT
YERS001	*Y. enterocolitica* 9610	NN
YERS014	*Y. enterocolitica*	NN
YERS015	*Y. enterocolitica*	NN
YERS002	*Y. kristensenii* 33639	NN
YERS017	*Y. pestis* Nairobi	NN (caf-)
YERS018	*Y. pestis* PMB19	BT
YERS019	*Y. pestis* Pestoides B	BT
YERS020	*Y. pestis* Pestoides F	NN (pla-)
YERS021	*Y. pestis* Harbin 35	BT
YERS022	*Y. pestis* Java 9	NN (caf-)
YERS023	*Y. pestis* CO92	BT
YERS059	*Y. pestis* CO92 pgm-	NN
YERS061	*Y. pestis* Kim10	BT
YERS008	*Y. pseudotuberculosis* 6904	NN
YERS012	*Y. ruckeri* 29473	NN

#### Linearity

**Figure 2:**
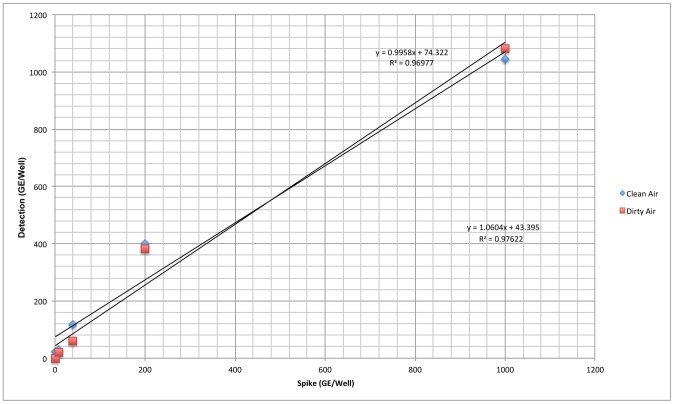
Linearity of response of the biothreat assay. *B. anthracis* DNA was spiked into AE buffer (Clean) or an extract from an environmental air filter (Dirty) at concentrations ranging from 0–1000 GE/well. The reported response from the PLEX-ID system was linear over the entire concentration range tested in both cases.

The ability of an instrument to provide measurements that are directly proportional to the concentration of the test analyte is referred to as linearity. Data obtained from experiments used to determine the LOD within an environmental matrix were analyzed for linearity. The total GE reported by the PLEX-ID was plotted against the actual concentration; linear trend lines were generated to determine linearity within the challenge concentrations. Figure 2 shows results for *B. anthracis* linearity test, demonstrating linearity for the entire range of concentrations tested. Other organisms were linear up to the maximum concentration tested. The data are summarized in Table 7.

**Table 7:** Linearity.

Organism	Range tested (GE/well)					Linear range (GE/well)
*Bacillus anthracis*	1000	200	40	8	2	0–1000
Vaccinia virus	5000	1000	200	40	8	0–5000
*Francisella tularensis*	1000	200	40	8	2	0–200
*Clostridium botulinum*	1000	200	40	8	2	0–200
*Y. pestis*	1000	200	40	8	2	40–1000
*Brucella melitensis* 1	1000	200	40	8	2	0–200
*Vibrio cholerae*	1000	200	40	8	2	0–200
*Burkholderia mallei*	1000	200	40	8	2	40–1000
*Salmonella enterica*	1000	200	40	8	2	8–40
*Clostridium perfringens*	1000	200	40	8	2	8–1000
*E. coli* O157:H7	1000	200	40	8	2	200–1000
*Shigella flexneri*	1000	200	40	8	2	8–1000
*Coxiella burnetii*	1000	200	40	8	2	40–1000
*Rickettsia prowazekii*	1000	200	40	8	2	8–200
VEE	1000	200	40	8	2	8–1000
Influenza	1000	200	40	8	2	Not tested

1 Only linear in samples without environmental air background.

## Discussion

The biothreat assay described here identifies ten bacterial and four viral biothreat clusters included in the NIAID priority pathogen (Category A: seven agents, Category B: 18; Category C: three) and HHS/USDA select agent (18 agents) lists. The assay also identifies a broad range of near-neighbor organisms that may cause severe disease in humans or animals or that may be harmless environmental organisms. The biothreat cluster analysis strategy using PCR/ESI-MS addresses several fundamental design requirements for biothreat protection. First, biothreat agents and near-neighbor organisms are identified unambiguously and equally. Closely related organisms often cause false alarms in conventional PCR approaches to detect biothreat agents. Perhaps more importantly, it enables identification of unexpected pathogens within the biothreat clusters that might be used in a biological attack. Second, the genetic targets for amplification in the biothreat assay are universally conserved, essential to microbial life. This lowers the risk of failed detections because these targets cannot be dispensed with by the microbe and would be difficult to modify by engineering to avoid detection. Third, the comprehensive nature of the biothreat assay enables very broad surveillance of the potential biothreat landscape, including the detection of the virulence plasmids where appropriate. Fourth, the PCR/ESI-MS instrumentation enables very high-throughput sample analysis; the theoretical maximum throughput of the biothreat assay on a current-generation PLEX-ID instrument is approximately 180 specimens over 24 hours.

Some of the component primer pairs of the comprehensive biothreat assay were validated before the biothreat assay was assembled. The *Orthopoxvirus* primers have been shown to detect and identify a diverse collection of over 30 isolates of orthopoxviruses and to identify sub-species and characterize viruses from the blood of rabbitpox-infected rabbits [11]. The *Alphavirus* primers were used to amplify a panel of 36 virus isolates representing characterized Old World and New World *Alphavirus* [12]. Base compositions from the resulting amplicons were used to unambiguously determine the species or subtypes of 35 of the isolates. In addition, the assay was used to identify *Alphavirus* directly in mosquitoes and detected an unanticipated Mucambo virus species [12].

The *Francisella* biocluster primers were used in an investigation to understand why the U.S. Government Biowatch sensors were triggered by an apparent false alarm on September 24–25, 2005 during a large public gathering along the Capital Mall area in Washington, DC. The sensors signaled low-level detections of *F. tularensis*. Uncertainty as to whether or not there was a biological attack led the CDC to issue an official national health advisory via the health alert network to alert healthcare personnel to possible tularemia exposure (CDCHAN-00238-05-09-30-ADV-N). Specimens analyzed by the primer pairs that comprise the *F. tularensis* biothreat cluster component of the biothreat assay were used to analyze the air specimens. The results showed that the base composition signature in the extracted air samples had the signature of *F. tularensis* subsp. *novicida*, a naturally occurring organism with significantly lower virulence than *F. tularensis* subsp. *tularensis*, the main bioagent in the cluster [46]. Thus the detections of *F. tularensis* observed in these air samples most likely represented detection of a naturally occurring organism. The biothreat assay described here would immediately identify this organism as a near neighbor of a biothreat agent.

The *V. cholerae* biocluster primers were previously field-tested using natural water samples from both freshwater lakes and the Georgian coastal zone of the Black Sea. Of the 278 total water samples screened, nine different *Vibrio* species were detected, 114 samples were positive for *V. cholerae*, and five samples were positive for the cholera toxin A gene (*ctxA*) [14]. The results were confirmed with conventional PCR.

The *Y. pestis* primers were used to identify the first reported case of plague in Afghanistan where the illness is associated with consumption of camel meat [47]. In late December 2007, an outbreak of acute gastroenteritis occurred in Nimroz Province of southern Afghanistan. Of the 83 patients, 17 died. Molecular testing of patient clinical samples and of tissue from the camel using PCR/ESI-MS revealed DNA signatures consistent with *Y. pestis*. Confirmatory testing using real-time PCR and immunological seroconversion of one of the patients confirmed that the outbreak was caused by plague with a rare gastrointestinal presentation.

Assembly of this comprehensive collection of biothreat cluster primers into a single assay on the PLEX-ID has the potential to serve a variety of biosecurity needs. First, the biothreat assay can be used for environmental surveillance − the application that was experimentally demonstrated in this manuscript. Another potential use of the assay is as a reflex test to the pan-bacterial PLEX-ID assay intended to identify all bacteria in normally sterile bodily fluids (e.g., blood, cerebral spinal fluid) [6]. In this concept of operation, the pan-bacterial assay would be used routinely in clinical diagnostics and, if potential biothreat agents were detected, the biothreat assay would be used to provide detailed analysis.

Although several of the primer pairs used in this assay were studied previously, the biothreat assay is a multiplexed version: Thirty-six primer pairs are combined into 16 PCR reactions. The multiplexed assay was tested with environmental air from Biowatch filters. Feasibility studies were performed at 1000 GE of each target organism spiked into background matrix. The environmental background did not inhibit the ability of PLEX-ID to correctly detect the target organisms. Additional studies were conducted to demonstrate the analytical performance of the assay. These included sensitivity, specificity, linearity, and breadth of coverage of the biothreat clusters. Analytical sensitivity of the target organisms varied between 40–1000 GE/well with no false positive detections, and false negative rates below 5% for most organisms tested. The matrix that was used as background had high loads of environmental bacterial signatures (data not shown), including alphaproteobacteria such as *Rickettsia* and *Mesorhizobium* species. These might account for the observed higher false positive rates for rickettsial species compared to other organisms. Breadth of coverage and specificity for detection of biothreat agents were determined with Critical Reagents Program (CRP; http://www.jpeocbd.osd.mil/packs/Default.aspx?pg=1205) reagents. There was an excellent correlation between PLEX-ID identifications and the identity of the spiked strains. Presence of two- to five- fold excess of a near-neighbor organism did not interfere with the detection of the biothreat agent. PLEX-ID assays in general are semi-quantitative at best and have a limited dynamic range for reporting genome levels of detected organisms [4]. The levels of organisms determined using the biothreat assay indicate the approximate genome equivalents/well for the target organism based on relative amplification compared to the calibrant. At higher target organism concentrations, the calibrant is often outcompeted in the PCR reaction, therefore the reported levels at the higher loads were inaccurate. This, however, does not interfere with the ability of the assay to detect the threat agent. In contrast, a specific RT-PCR assay targeting a single organism might be able to provide a measurement over a much larger linear range.

In summary, the PLEX-ID Biothreat Assay kit was evaluated for detection of biothreat agents in environmental air samples. The data presented demonstrate the capability of the PCR/ESI-MS method to accurately detect and identify organisms from ten bacterial and four viral biothreat clusters. The assay discriminated between target agent and near neighbors with high specificity and sensitivity. The method accurately reported each organism with which it was challenged and accurately identified threat species as a threat; species and/or strains that are not considered a threat were not reported as such. The assay is capable of simultaneous detection of most NIAID Category A, B, and C priority pathogens and HHS/USDA select agents and thus provides a means for comprehensive coverage using a high-throughput assay. The validation data support use of the Ibis PLEX-ID and the biothreat assay for detection of biological warfare agents in complex environmental matrices. Additional testing of this assay with an EU validation panel is described in a companion manuscript (Grunow et al. [49]).

## Materials and Methods

### Sample Preparation

The nucleic acid samples used in this study were obtained from the Critical Reagents Program (CRP), BEI Resources, ATCC, Keim Genetics Lab, or were prepared from MRI culture collections. Environmental collections were obtained on dry filter unit (DFU) filters from a variety of locations in the Washington, D.C. region. Nucleic acids were eluted from the environmental matrix by placing DFUs in 20 ml PBS/0.2% Triton X-200 and manually shaking. The eluent was then shaken in a Biospec Mini BeadBeater-96 with ATL buffer (Qiagen), Herring Sperm DNA, Proteinase K, and antifoam for 5 min. Samples were incubated for 21 min at 16°C and then centrifuged. AL buffer (Qiagen) was added to the supernatants and incubated for 1 min at 70°C. Ethanol was added to the sample, which was then loaded onto a Qiagen spin column, centrifuged, and sequentially washed with AL, AW2, and AW4 buffers (Qiagen). The sample was eluted from the spin columns in 200 µl of AE buffer (Qiagen).

### Assay Design

The primer pairs that make up the biothreat assay (Table 1) were selected to provide the desired resolving capability for the assay as described in detail in the Results section. Thirty-six primer pairs were used in the assay; primer pairs were grouped two to three per well to occupy 16 wells of a 96-well microtiter plate. The groupings were chosen to eliminate primer interactions and target overlap. The assay layout is shown in Figure S1. Each assay plate can be used to screen up to six samples.

### PCR and RT-PCR

Internal positive controls similar to the amplicon expected from one of the primer pairs in each of the multiplexed reactions were made from cloned synthetic DNA (BlueHeron Biotechnology, Bothell, WA) and were included in each PCR reaction at 100 copies per reaction. The internal controls were designed to be identical to the expected target priming regions with the exception of five-base pair deletions to enable the control to be distinguished from the target-derived amplicon. PCR was performed in a 50 µL reaction volume containing 5 µL nucleic acid extract in a reaction mix as previously described [48]. The plate was heat-sealed with foil on a Thermo Scientific ALPS microplate heat sealer (Rockford, IL). Each sealed plate was loaded onto a Mastercycler Pro thermocycler (Eppendorf, Hauppauge, NY) and PCR-amplified under the following conditions: 95°C for 10 min; then 8 cycles of 95°C for 30 s, 48°C for 30 s, and 72°C for 30 s; then 37 cycles of 95°C for 15 s, 56°C for 20 s, and 72°C for 20 s; followed by 72°C for 2 min, and 99°C for 20 min. One-step RT-PCR was performed in wells with primers designed for viral detection. Since all reactions for a sample were run in the same 96-well plate RT-PCR cycling conditions were used for both the RT-PCR and PCR reactions as previously described [12].

### Target Detection

After thermocycling, plates were stored at −40°C until the samples could be analyzed by ESI/MS on the PLEX-ID. Mass spectrometry was performed on a PLEX-ID biosensor (Abbott Molecular, Des Plaines, IL). After PCR amplification, 30 µL aliquots of each PCR reaction were desalted and analyzed by mass spectrometry as previously described [2], [4]. Briefly, the PLEX-ID platform is capable of analyzing nearly 3000 PCR reactions in 24 hours and integrates a novel sample purification scheme with a high throughput fluidics/robotics platform. The PLEX-ID instrument is comprised of an input plate stacker which accommodates fifteen 96-well microtiter plates, an automated purification module which desalts and purifies amplicons with a magnetic-bead-based weak anion exchange method, and an autosampler coupled to a novel dual electrospray head which injects analytes into a Perkin Elmer ESI-TOF mass spectrometer. The platform analyzes one PCR reaction every 30 seconds in a fully automated modality. The PLEX-ID platform utilizes a novel carousel-based design for the rapid and efficient purification of PCR amplicons. The carousel is comprised of 22 identical spin cuvette modules in which the analyte solution is purified prior to ESI-MS analysis. Every 30 seconds the carousel rotates by one position facilitating the aspiration/or dispensation of the requisite reagents.

### Data Analysis and Results Reporting

Data analysis and results reporting was performed in an automated fashion using on-board computer on the Ibis PLEX-ID system. For this assay, a customized reporting rule set was designed that allowed rapid and accurate detection of the biothreat targets. The biothreat assay report has 21 primer groups or threat clusters as shown in Figure S2. These groups consist of primer pairs used to identify the target biothreat organisms. Each of the threat clusters is treated independently and the results are reported for each cluster separately. Thus, the presence or absence of each of the target biothreat clusters is directly reported. Mixed detections of two or more threats or a threat with an unrelated near neighbor in another group are also reported. Further, two additional metrics (Q-score and level) are provided in the report to assess the quality of the reported detection. The Q-Score is a rating between 0 (low) and 1 (high) of the confidence in the identification of the organism. The Q-Score is based on a number of different parameters such as, the multi-primer joint log likelihood ratio, which is an indicator of how well the hypothesized organisms as a group represent the observed data; the multi-primer single log likelihood ratio, which is an indicator of how significant the contribution of a single organism is to the solution; the fraction of missed detections, which represents the percentage of primers for a detected organism that should have produced known base count compositions, but did not; and, finally, the fraction of no data, which indicates the percentage of primers for a detected organism for which no known data exists within the PLEX-ID system. For the biothreat assay described here, a Q-score ≥0.85 is considered a reportable result. The level is an indication of the amount of the amplicon present in the sample reported as genome equivalents/well. This is calculated with reference to the internal calibrant as described previously [4]. The normal range for reporting these levels is between 0.1× and 10× the levels of internal controls in the assay, which in the case of the PLEX-ID Biothreat assay represents a working range of ∼10 GE/well to 1000 GE/well.

In addition to the summary style report shown in Figure S2, the system is capable of reporting organism/strain level matches based on the genomic sequence data in the PLEX-ID database (Figure S3). This is provided as a research utility tool in a separate analysis workstation. Additional details for each of the matches, including the detected base compositions and levels, are available using this report.

## Supporting Information

Figure S1 **Biothreat assay plate layout. Left panel:** Sample wells and target biothreat cluster for each primer pair are indicated. Each well contains two or three multiplexed primer pairs. Letters A through H represent 8 rows of a 96-well plate, whereas numbers 1 through 12 represent the columns. Each sample is analyzed in 16 PCR reaction wells and six samples can be tested per PCR plate. Details of the primer pairs are given in Table 1. **Right panel:** The 96-well PCR plate layout. Each PCR well includes a synthetic nucleic acid template that serves as a calibrant. In multiplexed wells, this calibrant provides an amplicon similar to the amplicon expected for the organism shown in red.
(DOCX)

Figure S2 **Example of PLEX-ID summary report.** Each biothreat cluster is listed separately. Detection of an organism within a cluster is listed at the species level. If no organism within the cluster is detected, the cluster is marked as “Not Detected”. The plasmid markers are listed separately.
(DOCX)

Figure S3 **Example of PLEX-ID detailed report.** Base compositions associated with each detection are reported. The markers (pXO1 and pXO2 in this example) are reported independently of the biothreat cluster (*Bacillus* in this case).
(DOCX)

Figure S4 **Limits of detection for the biothreat assay. Left panel:** Analytical limits of the multiplexed primer pairs using synthetic DNA/RNA constructs. **Bottom left panel:** Detection of spiked DNA/RNA in AE buffer. **Bottom right panel:** Detection of spiked DNA/RNA in “Dirty Air”. The requirement for LOD reporting was detection in all the primer pairs for any given target. Highlighted cells show the concentration at which detection of no more than one replicate was missed.
(DOCX)

Table S1 **USAMRIID B. anthracis strains used in the study.**
(DOCX)

Table S2 **Base composition signatures for Keim Genetics Lab Bacillus collection.** The signatures that are bolded or italicized indicate a SNP variation compared to the predominant signature.
(DOCX)

Table S3 **Base composition signatures for Keim Genetics Lab Bacillus collection Clade A1a genotypes.**
(DOCX)

Table S4 **Expected** ***Yersinia pestis*** **genomic signatures and near-neighbor organism signatures.**
(DOCX)

Table S5 **Experimental data on** ***Yersinia pestis*** **from the USAMRIID Collection.**
(DOCX)

Table S6 **Experimental data on** ***Yersinia pestis*** **near neighbors.**
(DOCX)

Table S7 ***Francisella tularensis*** **signatures from genome sequence data and experimental measurements.**
(DOCX)

Table S8 **Expected** ***Vibrio*** **species signatures.**
(DOCX)

Table S9 **Expected** ***Burkholderia*** **species signatures.**
(DOCX)

Table S10 **Expected** ***Brucella*** **species signatures.**
(DOCX)

Table S11 **Expected** ***Clostridium*** **species signatures.**
(DOCX)

Table S12 **Expected** ***Coxiella*** **species signatures.**
(DOCX)

Table S13 **Expected** ***Rickettsia*** **species signatures.**
(DOCX)

Table S14 **Expected** ***Enterobacteriaceae*** **species signatures.**
(DOCX)

Table S15 **Expected** ***Shigella species*** **signatures.**
(DOCX)

Table S16 **Expected** ***Alphavirus*** **signatures.**
(DOCX)

Table S17 **Expected** ***Orthopoxvirus*** **signatures.**
(DOCX)

Table S18 **Expected Influenza A virus (H5N1) signatures and potential overlap with non-H5N1 species.**
(DOCX)

Table S19 **Expected** ***Filovirus*** **signatures.**
(DOCX)
