# Supplementary figures and images for: Comprehensive Biothreat Cluster Identification by PCR/Electrospray-Ionization Mass Spectrometry
Source: PLoS One. 2012 Jun 29;7(6):e36528. doi: 10.1371/journal.pone.0036528 (PMC3387173; doi:10.1371/journal.pone.0036528)

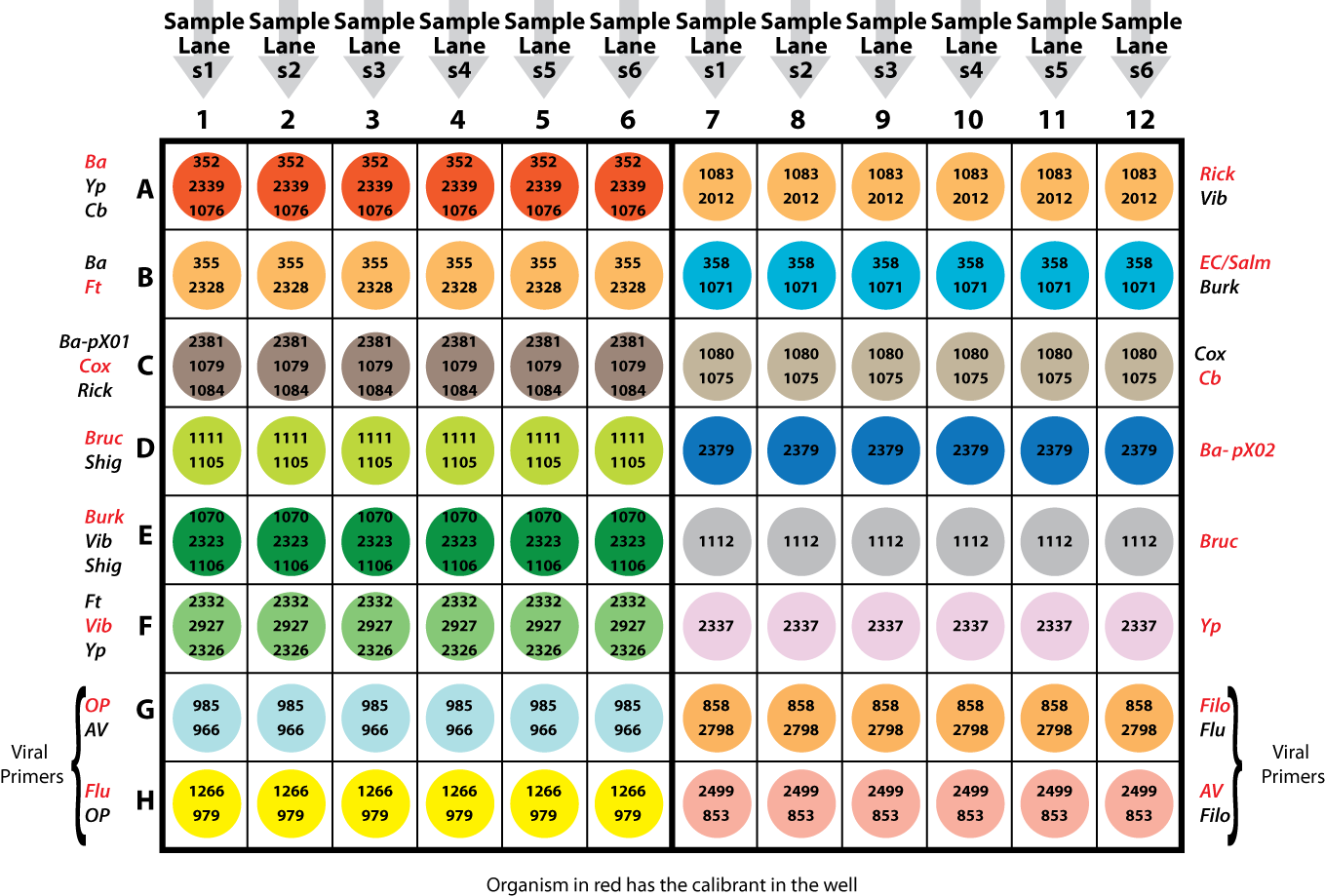


**Figure S1. *Biothreat assay plate layout.***

Supplement: Figure S1 — Biothreat assay plate layout. Left panel: Sample wells and target biothreat cluster for each primer pair are indicated. Each well contains two or three multiplexed primer pairs. Letters A through H represent 8 rows of a 96-well plate, whereas numbers 1 through 12 represent the columns. Each sample is analyzed in 16 PCR reaction wells and six samples can be tested per PCR plate. Details of the primer pairs are given in Table 1. Right panel: The 96-well PCR plate layout. Each PCR well includes a synthetic nucleic acid template that serves as a calibrant. In multiplexed wells, this calibrant provides an amplicon similar to the amplicon expected for the organism shown in red. (DOCX) [file pone.0036528.s001.docx]

| 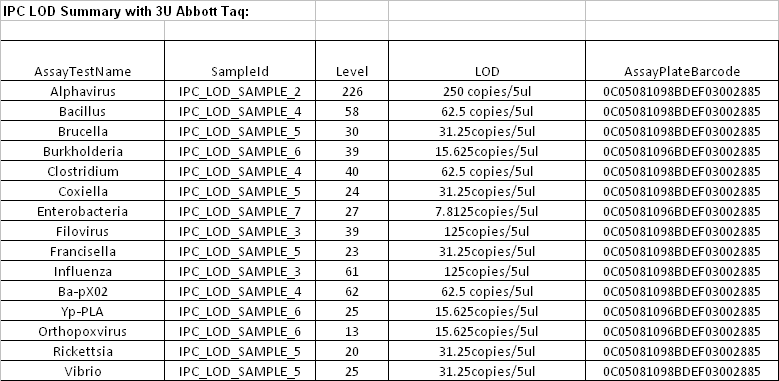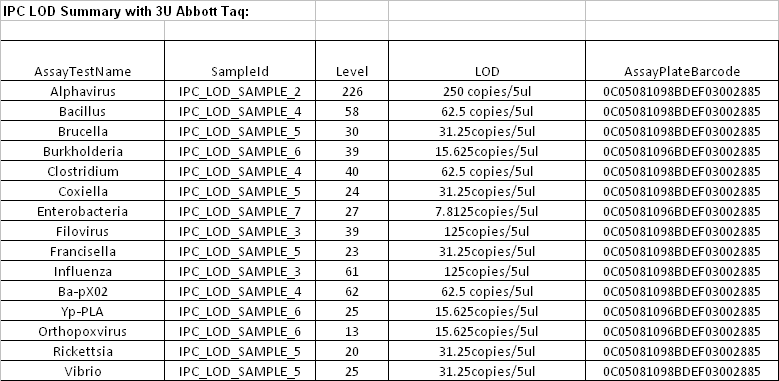 |  |
| --- | --- |
|  |  |

**Figure S4. *Limits of detection for the biothreat assay.***

Supplement: Figure S4 — Limits of detection for the biothreat assay. Left panel: Analytical limits of the multiplexed primer pairs using synthetic DNA/RNA constructs. Bottom left panel: Detection of spiked DNA/RNA in AE buffer. Bottom right panel: Detection of spiked DNA/RNA in “Dirty Air”. The requirement for LOD reporting was detection in all the primer pairs for any given target. Highlighted cells show the concentration at which detection of no more than one replicate was missed. (DOCX) [file pone.0036528.s004.docx]
