# Supplementary material for: Comprehensive Biothreat Cluster Identification by PCR/Electrospray-Ionization Mass Spectrometry
Source: PLoS One. 2012 Jun 29;7(6):e36528. doi: 10.1371/journal.pone.0036528 (PMC3387173; doi:10.1371/journal.pone.0036528)
Supplement: Figure S2 — Example of PLEX-ID summary report. Each biothreat cluster is listed separately. Detection of an organism within a cluster is listed at the species level. If no organism within the cluster is detected, the cluster is marked as “Not Detected”. The plasmid markers are listed separately. (DOCX) [file pone.0036528.s002.docx]

**Figure S2. *Example of PLEX-ID summary report.***
