# Supplementary material for: Comprehensive Biothreat Cluster Identification by PCR/Electrospray-Ionization Mass Spectrometry
Source: PLoS One. 2012 Jun 29;7(6):e36528. doi: 10.1371/journal.pone.0036528 (PMC3387173; doi:10.1371/journal.pone.0036528)
Supplement: Figure S3 — Example of PLEX-ID detailed report. Base compositions associated with each detection are reported. The markers (pXO1 and pXO2 in this example) are reported independently of the biothreat cluster (Bacillus in this case). (DOCX) [file pone.0036528.s003.docx]

**Figure S3. *Example of PLEX-ID detailed report.***
