# Supplementary material for: Comprehensive Biothreat Cluster Identification by PCR/Electrospray-Ionization Mass Spectrometry
Source: PLoS One. 2012 Jun 29;7(6):e36528. doi: 10.1371/journal.pone.0036528 (PMC3387173; doi:10.1371/journal.pone.0036528)
Supplement: Table S1 — USAMRIID B. anthracis strains used in the study. (DOCX) [file pone.0036528.s005.docx]

Table S1: USAMRIID *B. anthracis* strains used in the study

| **Sample Number** | **Organism** | **ATCC #** | **Product #** | **"Common Name"/virulence factors** | **Expected Plasmid Profile** |
| --- | --- | --- | --- | --- | --- |
| 1 | CONTROL *Bacillus anthracis* | NA | BACI008-BZ021 | BA1004 Ames (PA+ ,CapB+) | *pXO1 + , pXO2 +* |
| 2 | *Bacillus anthracis* | NA | BACI002-DP15 | BA0068 V770-NP1R (PA +,CapB- ) | *pXO1 + , pXO2 -* |
| 3 | *Bacillus anthracis* | NA | BACI003-DP14 | BA0070 Delta NH-1(PA- ,CapB+) | *pXO1 - , pXO2 +* |
| 4 | *Bacillus anthracis* | NA | BACI004-DP13 | BA0074 STI (PA+ ,CapB-) | *pXO1 + , pXO2 -* |
| 5 | *Bacillus anthracis* | NA | BACI006-DP10 | BA0078 New Hampshire (PA+ ,CapB+) | *pXO1 + , pXO2 +* |
| 6 | *Bacillus anthracis* | NA | BACI007-BZ016 | BA1000 Vollum (PA+, CapB+) | *pXO1 + , pXO2 +* |
| 7 | *Bacillus anthracis* | NA | BACI012-BZ007 | BA1036 Sterne (PA +, CapB- ) | *pXO1 + , pXO2 -* |
| 8 | *Bacillus anthracis* | NA | BACI013-BZ024 | BA1176 (PA+, CapB+ ) | *pXO1 + , pXO2 +* |
| 9 | *Bacillus anthracis* | NA | BACI014-BZ030 | (PA+ ,CapB- ) | *pXO1 + , pXO2 -* |
| 10 | *Bacillus cereus* | 10876 | BACI015-BZ020 |  |  |
| 11 | *Bacillus cereus* | 13061 | BACI016-DP11 |  |  |
| 12 | *Bacillus coagulans* | 7050 | BACI020-DP15 |  |  |
| 13 | *Bacillus licheniformis* | 12759 | BACI021-BZ008 |  |  |
| 14 | *Bacillus macerans (Paenibacillus macerans)* | 8244 | BACI025-DP11 | BA1073 |  |
| 15 | *Bacillus megaterium* | NA | BACI026-DP13 | BA1070 |  |
| 16 | *Bacillus mycoides* | 31101 | BACI027-QM038 |  |  |
| 17 | *Bacillus polymyxa* | 842 | BACI030-BZ010 | BA0103 |  |
| 18 | *Bacillus sphaericus* | 4525 | BACI031-BZ011 |  |  |
| 19 | *Bacillus subtilis var niger* | 6633 | BACI033-BZ022 |  |  |
| 20 | *Bacillus subtilis var niger* | 9372 | BACI034-BZ025 | BA1102 |  |
| 21 | *Bacillus thuringiensis* | 39152 | BACI037-BZ029 |  |  |
| 22 | *Bacillus popilliae* | 14706 | BACI050-DP10 |  |  |
| 23 | *Bacillus subtilis var. niger* | NA | BACI051-DP14 | BA0057 |  |
| 24 | *Bacillus thuringiensis* | NA | BACI052-BZ028 | BA0086 |  |
| 25 | *Bacillus anthracis* | 4728 | BACI055-BZ034 | Pasteur-like (PA-,CapB+) | *pXO1 - , pXO2 +* |
| 26 | *Bacillus anthracis* | NA | BACI056-BZ031 | Delta Sterne (PA-,CapB-) | *pXO1 - , pXO2 -* |
| 27 | *Bacillus mycoides* | 21929 | BACI088-QM039 |  |  |
| 28 | *Bacillus cereus* | 10987 | BACI103-QM036 |  |  |
| 29 | *Bacillus anthracis* | NA | BACI124-QM012 | BA1002 Vollum 1b (PA+, CapB+) | *pXO1 + , pXO2 +* |
| 30 | *Bacillus halodurans* | 21591 | BACI207D-ATCC |  |  |
| 31 | *Bacillus (Brevibacillus) brevis* | 8246 | BACI208-QM037 |  |  |
| 32 | *Bacillus sp Ba813 #11 9594/3* |  | BACI209-QM040 |  |  |
