# Supplementary material for: Comprehensive Biothreat Cluster Identification by PCR/Electrospray-Ionization Mass Spectrometry
Source: PLoS One. 2012 Jun 29;7(6):e36528. doi: 10.1371/journal.pone.0036528 (PMC3387173; doi:10.1371/journal.pone.0036528)
Supplement: Table S2 — Base composition signatures for Keim Genetics Lab Bacillus collection. The signatures that are bolded or italicized indicate a SNP variation compared to the predominant signature. (DOCX) [file pone.0036528.s006.docx]

Table S2: Base composition signatures for Keim Genetics Lab *Bacillus* collection. The signatures that are bolded or italicized indicate a SNP variation compared to the predominant signature

| **Sample Name** | **Bacillus_INFB (BCT352)** | **Bacillus_SSPE (BCT355)** | **BA_pXO1 (BCT2381)** | **BA_pXO2 (BCT2379)** |
| --- | --- | --- | --- | --- |
| A0402 | A34 G25 C21 T25 | A42 G23 C23 T21 | A41 G15 C22 T34 | A44 G27 C14 T41 |
| A0442 | A34 G25 C21 T25 | A42 G23 C23 T21 | A41 G15 C22 T34 | A44 G27 C14 T41 |
| A0488 | A34 G25 C21 T25 | A42 G23 C23 T21 | A41 G15 C22 T34 | A44 G27 C14 T41 |
| A0071 | A34 G25 C21 T25 | **A41 G24 C23 T21** | A41 G15 C22 T34 | A44 G27 C14 T41 |
| A0001 | A34 G25 C21 T25 | A42 G23 C23 T21 | A41 G15 C22 T34 | A44 G27 C14 T41 |
| A0026 | A34 G25 C21 T25 | A42 G23 C23 T21 | A41 G15 C22 T34 | A44 G27 C14 T41 |
| A0032 | A34 G25 C21 T25 | A42 G23 C23 T21 | A41 G15 C22 T34 | A44 G27 C14 T41 |
| A0033 | A34 G25 C21 T25 | A42 G23 C23 T21 | A41 G15 C22 T34 | A44 G27 C14 T41 |
| A0034 | A34 G25 C21 T25 | A42 G23 C23 T21 | A41 G15 C22 T34 | A44 G27 C14 T41 |
| A0035 | A34 G25 C21 T25 | A42 G23 C23 T21 | A41 G15 C22 T34 | A44 G27 C14 T41 |
| A0038 | A34 G25 C21 T25 | A42 G23 C23 T21 | A41 G15 C22 T34 | A44 G27 C14 T41 |
| A0048 | A34 G25 C21 T25 | A42 G23 C23 T21 | A41 G15 C22 T34 | A44 G27 C14 T41 |
| A0049 | A34 G25 C21 T25 | A42 G23 C23 T21 | A41 G15 C22 T34 | A44 G27 C14 T41 |
| A0062 | A34 G25 C21 T25 | A42 G23 C23 T21 | Target Absent | Target Absent |
| A0102 | A34 G25 C21 T25 | A42 G23 C23 T21 | A41 G15 C22 T34 | A44 G27 C14 T41 |
| A0104 | A34 G25 C21 T25 | A42 G23 C23 T21 | A41 G15 C22 T34 | A44 G27 C14 T41 |
| A0148 | A34 G25 C21 T25 | A42 G23 C23 T21 | A41 G15 C22 T34 | A44 G27 C14 T41 |
| A0149 | A34 G25 C21 T25 | A42 G23 C23 T21 | A41 G15 C22 T34 | A44 G27 C14 T41 |
| A0152 | A34 G25 C21 T25 | A42 G23 C23 T21 | A41 G15 C22 T34 | A44 G27 C14 T41 |
| A0158 | A34 G25 C21 T25 | A42 G23 C23 T21 | A41 G15 C22 T34 | A44 G27 C14 T41 |
| A0168 | A34 G25 C21 T25 | **A41 G24 C23 T21** | A41 G15 C22 T34 | A44 G27 C14 T41 |
| A0172 | A34 G25 C21 T25 | **A41 G24 C23 T21** | A41 G15 C22 T34 | A44 G27 C14 T41 |
| A0174 | A34 G25 C21 T25 | **A41 G24 C23 T21** | A41 G15 C22 T34 | A44 G27 C14 T41 |
| A0186 | A34 G25 C21 T25 | A42 G23 C23 T21 | A41 G15 C22 T34 | A44 G27 C14 T41 |
| A0188 | A34 G25 C21 T25 | A42 G23 C23 T21 | A41 G15 C22 T34 | A44 G27 C14 T41 |
| A0193 | A34 G25 C21 T25 | **A41 G24 C23 T21** | A41 G15 C22 T34 | A44 G27 C14 T41 |
| A0196 | A34 G25 C21 T25 | A42 G23 C23 T21 | A41 G15 C22 T34 | A44 G27 C14 T41 |
| A0220 | A34 G25 C21 T25 | A42 G23 C23 T21 | A41 G15 C22 T34 | A44 G27 C14 T41 |
| A0231 | A34 G25 C21 T25 | A42 G23 C23 T21 | A41 G15 C22 T34 | A44 G27 C14 T41 |
| A0236 | A34 G25 C21 T25 | A42 G23 C23 T21 | A41 G15 C22 T34 | A44 G27 C14 T41 |
| A0241 | A34 G25 C21 T25 | A42 G23 C23 T21 | A41 G15 C22 T34 | Target Absent |
| A0242 | A34 G25 C21 T25 | A42 G23 C23 T21 | A41 G15 C22 T34 | A44 G27 C14 T41 |
| A0245 | A34 G25 C21 T25 | A42 G23 C23 T21 | A41 G15 C22 T34 | A44 G27 C14 T41 |
| A0256 | A34 G25 C21 T25 | A42 G23 C23 T21 | A41 G15 C22 T34 | A44 G27 C14 T41 |
| A0264 | A34 G25 C21 T25 | A42 G23 C23 T21 | A41 G15 C22 T34 | A44 G27 C14 T41 |
| A0267 | A34 G25 C21 T25 | A42 G23 C23 T21 | A41 G15 C22 T34 | Target Absent |
| A0274 | A34 G25 C21 T25 | A42 G23 C23 T21 | A41 G15 C22 T34 | A44 G27 C14 T41 |
| A0280 | A34 G25 C21 T25 | A42 G23 C23 T21 | A41 G15 C22 T34 | A44 G27 C14 T41 |
| A0286 | A34 G25 C21 T25 | A42 G23 C23 T21 | A41 G15 C22 T34 | A44 G27 C14 T41 |
| A0293 | A34 G25 C21 T25 | A42 G23 C23 T21 | A41 G15 C22 T34 | A44 G27 C14 T41 |
| A0300 | A34 G25 C21 T25 | **A41 G24 C23 T21** | A41 G15 C22 T34 | A44 G27 C14 T41 |
| A0308 | A34 G25 C21 T25 | **A41 G24 C23 T21** | A41 G15 C22 T34 | A44 G27 C14 T41 |
| A0324 | A34 G25 C21 T25 | A42 G23 C23 T21 | A41 G15 C22 T34 | A44 G27 C14 T41 |
| A0328 | A34 G25 C21 T25 | A42 G23 C23 T21 | A41 G15 C22 T34 | A44 G27 C14 T41 |
| A0330 | A34 G25 C21 T25 | A42 G23 C23 T21 | A41 G15 C22 T34 | A44 G27 C14 T41 |
| A0331 | A34 G25 C21 T25 | A42 G23 C23 T21 | A41 G15 C22 T34 | A44 G27 C14 T41 |
| A0336 | A34 G25 C21 T25 | A42 G23 C23 T21 | A41 G15 C22 T34 | A44 G27 C14 T41 |
| A0337 | A34 G25 C21 T25 | A42 G23 C23 T21 | A41 G15 C22 T34 | A44 G27 C14 T41 |
| A0341 | A34 G25 C21 T25 | A42 G23 C23 T21 | A41 G15 C22 T34 | A44 G27 C14 T41 |
| A0343 | A34 G25 C21 T25 | A42 G23 C23 T21 | A41 G15 C22 T34 | A44 G27 C14 T41 |
| A0344 | A34 G25 C21 T25 | A42 G23 C23 T21 | A41 G15 C22 T34 | A44 G27 C14 T41 |
| A0352 | A34 G25 C21 T25 | A42 G23 C23 T21 | A41 G15 C22 T34 | A44 G27 C14 T41 |
| A0353 | A34 G25 C21 T25 | A42 G23 C23 T21 | A41 G15 C22 T34 | A44 G27 C14 T41 |
| A0354 | A34 G25 C21 T25 | A42 G23 C23 T21 | A41 G15 C22 T34 | Target Absent |
| A0359 | A34 G25 C21 T25 | A42 G23 C23 T21 | A41 G15 C22 T34 | A44 G27 C14 T41 |
| A0361 | A34 G25 C21 T25 | A42 G23 C23 T21 | A41 G15 C22 T34 | A44 G27 C14 T41 |
| A0362 | A34 G25 C21 T25 | A42 G23 C23 T21 | A41 G15 C22 T34 | A44 G27 C14 T41 |
| A0363 | A34 G25 C21 T25 | A42 G23 C23 T21 | A41 G15 C22 T34 | A44 G27 C14 T41 |
| A0365 | A34 G25 C21 T25 | *A43 G22 C23 T21* | A41 G15 C22 T34 | A44 G27 C14 T41 |
| A0369 | A34 G25 C21 T25 | **A41 G24 C23 T21** | A41 G15 C22 T34 | A44 G27 C14 T41 |
| A0371 | A34 G25 C21 T25 | A42 G23 C23 T21 | A41 G15 C22 T34 | A44 G27 C14 T41 |
| A0374 | A34 G25 C21 T25 | **A41 G24 C23 T21** | A41 G15 C22 T34 | Target Absent |
| A0376 | A34 G25 C21 T25 | A42 G23 C23 T21 | A41 G15 C22 T34 | A44 G27 C14 T41 |
| A0379 | A34 G25 C21 T25 | A42 G23 C23 T21 | A41 G15 C22 T34 | A44 G27 C14 T41 |
| A0380 | A34 G25 C21 T25 | A42 G23 C23 T21 | A41 G15 C22 T34 | A44 G27 C14 T41 |
| A0382 | A34 G25 C21 T25 | A42 G23 C23 T21 | A41 G15 C22 T34 | A44 G27 C14 T41 |
| A0388 | A34 G25 C21 T25 | A42 G23 C23 T21 | A41 G15 C22 T34 | A44 G27 C14 T41 |
| A0389 | A34 G25 C21 T25 | A42 G23 C23 T21 | A41 G15 C22 T34 | A44 G27 C14 T41 |
| A0392 | A34 G25 C21 T25 | **A41 G24 C23 T21** | A41 G15 C22 T34 | A44 G27 C14 T41 |
| A0404 | A34 G25 C21 T25 | A42 G23 C23 T21 | A41 G15 C22 T34 | A44 G27 C14 T41 |
| A0406 | A34 G25 C21 T25 | A42 G23 C23 T21 | A41 G15 C22 T34 | A44 G27 C14 T41 |
| A0412 | A34 G25 C21 T25 | A42 G23 C23 T21 | A41 G15 C22 T34 | A44 G27 C14 T41 |
| A0413 | A34 G25 C21 T25 | A42 G23 C23 T21 | A41 G15 C22 T34 | A44 G27 C14 T41 |
| A0415 | A34 G25 C21 T25 | A42 G23 C23 T21 | A41 G15 C22 T34 | A44 G27 C14 T41 |
| A0417 | A34 G25 C21 T25 | A42 G23 C23 T21 | A41 G15 C22 T34 | A44 G27 C14 T41 |
| A0419 | A34 G25 C21 T25 | A42 G23 C23 T21 | A41 G15 C22 T34 | A44 G27 C14 T41 |
| A0435 | A34 G25 C21 T25 | A42 G23 C23 T21 | A41 G15 C22 T34 | A44 G27 C14 T41 |
| A0446 | A34 G25 C21 T25 | A42 G23 C23 T21 | A41 G15 C22 T34 | A44 G27 C14 T41 |
| A0451 | A34 G25 C21 T25 | A42 G23 C23 T21 | A41 G15 C22 T34 | A44 G27 C14 T41 |
| A0460 | A34 G25 C21 T25 | A42 G23 C23 T21 | A41 G15 C22 T34 | A44 G27 C14 T41 |
| A0461 | A34 G25 C21 T25 | A42 G23 C23 T21 | A41 G15 C22 T34 | A44 G27 C14 T41 |
| A0462 | A34 G25 C21 T25 | A42 G23 C23 T21 | **A40 G16 C22 T34** | **A45 G27 C13 T41** |
| A0463 | A34 G25 C21 T25 | A42 G23 C23 T21 | A41 G15 C22 T34 | A44 G27 C14 T41 |
| A0464 | A34 G25 C21 T25 | A42 G23 C23 T21 | A41 G15 C22 T34 | A44 G27 C14 T41 |
| A0465 | A34 G25 C21 T25 | A42 G23 C23 T21 | A41 G15 C22 T34 | A44 G27 C14 T41 |
| A0468 | A34 G25 C21 T25 | A42 G23 C23 T21 | A41 G15 C22 T34 | A44 G27 C14 T41 |
| A0489 | A34 G25 C21 T25 | A42 G23 C23 T21 | A41 G15 C22 T34 | A44 G27 C14 T41 |
| A0593 | A34 G25 C21 T25 | A42 G23 C23 T21 | Target Absent | A44 G27 C14 T41 |
| A1055 | A34 G25 C21 T25 | A42 G23 C23 T21 | Target Absent | A44 G27 C14 T41 |
