# Supplementary material for: Comprehensive Biothreat Cluster Identification by PCR/Electrospray-Ionization Mass Spectrometry
Source: PLoS One. 2012 Jun 29;7(6):e36528. doi: 10.1371/journal.pone.0036528 (PMC3387173; doi:10.1371/journal.pone.0036528)
Supplement: Table S3 — Base composition signatures for Keim Genetics Lab Bacillus collection Clade A1a genotypes. (DOCX) [file pone.0036528.s007.docx]

Table S3: Base composition signatures for Keim Genetics Lab *Bacillus* collection Clade A1a genotypes

| **Genotype** | **Sample Name^1^** | **Genotype^2^** | ***Bacillus*_INFB (BCT352)** | ***Bacillus*_SSPE (BCT355)** | **BA_pXO1 (BCT2381)** | **BA_pXO2 (BCT2379)** |
| --- | --- | --- | --- | --- | --- | --- |
| A1a | A0300 | 2 Canada 80-167C-5 ADRI K2284 | A34 G25 C21 T25 | **A41 G24 C23 T21** | A41 G15 C22 T34 | A44 G27 C14 T41 |
| A1a | A0174 | 3 Canada 74-412C-8 ADRI K7441 | A34 G25 C21 T25 | **A41 G24 C23 T21** | A41 G15 C22 T34 | A44 G27 C14 T41 |
| A1a | A0374 | 4 Iowa BA1007/#81 USAMRIID K8113 | A34 G25 C21 T25 | **A41 G24 C23 T21** | A41 G15 C22 T34 | A44 G27 C14 T41 |
| A1a | A0308 | 5 Canada 91-382C-1 ADRI K1081 | A34 G25 C21 T25 | **A41 G24 C23 T21** | A41 G15 C22 T34 | A44 G27 C14 T41 |
| A1a | A0392 | 6 Texas C93022281 TVMDL K2165 | A34 G25 C21 T25 | **A41 G24 C23 T21** | A41 G15 C22 T34 | A44 G27 C14 T41 |
| A1a | A0369 | 7 Canada BA0018 USAMRIID K8960 | A34 G25 C21 T25 | **A41 G24 C23 T21** | A41 G15 C22 T34 | A44 G27 C14 T41 |
| A1a | A0168 | 8 Canada 72-241C-A ADRI K1040 | A34 G25 C21 T25 | **A41 G24 C23 T21** | A41 G15 C22 T34 | A44 G27 C14 T41 |
| A1a | A0172 | 9 Canada 74-389C-52 ADRI K3897 | A34 G25 C21 T25 | **A41 G24 C23 T21** | A41 G15 C22 T34 | A44 G27 C14 T41 |
| A1a | A0193 | 10 S. Dakota 96-10355 ADRDL K1256 | A34 G25 C21 T25 | **A41 G24 C23 T21** | A41 G15 C22 T34 | A44 G27 C14 T41 |
