# Supplementary material for: Comprehensive Biothreat Cluster Identification by PCR/Electrospray-Ionization Mass Spectrometry
Source: PLoS One. 2012 Jun 29;7(6):e36528. doi: 10.1371/journal.pone.0036528 (PMC3387173; doi:10.1371/journal.pone.0036528)
Supplement: Table S4 — Expected Yersinia pestis genomic signatures and near-neighbor organism signatures. (DOCX) [file pone.0036528.s008.docx]

Table S4. Expected *Yersinia pestis* genomic signatures and near-neighbor organism signatures

| **Organism** | **Strain** | **Phenotype** | **GenBank ID** | **VALS_(BCT358)** | **YP_INV (BCT2326)** | **YP_PLA (BCT2337)** | **YP_CAF (BCT2339)** |
| --- | --- | --- | --- | --- | --- | --- | --- |
| *Y. pestis* | Antiqua | PLA+/CAF+ | gi\|108793532 | A26 G34 C35 T21 | A29 G21 C20 T23 | A21 G16 C18 T24 | A29 G20 C32 T31 |
| *Y. pestis* | CO-92 Biovar Orientalis | PLA+/CAF+ | gi\|16082781 | A26 G34 C35 T21 | A29 G21 C20 T23 | A21 G16 C18 T24 | A29 G20 C32 T31 |
| *Y. pestis* | D106004 | PLA+/CAF+ | gi\|262363886 | A26 G34 C35 T21 | A29 G21 C20 T23 | A21 G16 C18 T24 | A29 G20 C32 T31 |
| *Y. pestis* | D182038 | PLA+/CAF+ | gi\|262364042 | A26 G34 C35 T21 | A29 G21 C20 T23 | A21 G16 C18 T24 | A29 G20 C32 T31 |
| *Y. pestis* | KIM | PLA+/CAF+ | gi\|2996286 | A26 G34 C35 T21 | A29 G21 C20 T23 | A21 G16 C18 T24 | A29 G20 C32 T31 |
| *Y. pestis* | KIM5 P12 (Biovar Mediaevalis) | PLA+/CAF+ | gi\|31795183 | A26 G34 C35 T21 | A29 G21 C20 T23 | A21 G16 C18 T24 | A29 G20 C32 T31 |
| *Y. pestis* | Nepal516 | PLA+/CAF+ | gi\|108793732 | A26 G34 C35 T21 | A29 G21 C20 T23 | A21 G16 C18 T24 | A29 G20 C32 T31 |
| *Y. pestis* | Z176003 | PLA+/CAF+ | gi\|294501992 | A26 G34 C35 T21 | A29 G21 C20 T23 | A21 G16 C18 T24 | A29 G20 C32 T31 |
| *Y. pestis* | 91001 | PLA+/CAF+ | gi\|45439865 | A26 G34 C35 T21 | A29 G21 C20 T23 | A21 G16 C18 T24 | A29 G20 C32 T31 |
| *Y. pestis* | CA88-4125 | PLA+/CAF+ | gi\|149192721 | A26 G34 C35 T21 | A29 G21 C20 T23 | A21 G16 C18 T24 | A29 G20 C32 T31 |
| *Y. pestis* | CO92 | PLA+/CAF+ | gi\|15979723 | A26 G34 C35 T21 | A29 G21 C20 T23 | A21 G16 C18 T24 | A29 G20 C32 T31 |
| *Y. pestis* | Angola | PLA+/CAF- | gi\|162417821 | A26 G34 C35 T21 | A29 G21 C20 T23 | A21 G16 C18 T24 | Target Absent |
| *Y. pestis* | Pestoides F | PLA-/CAF+ | gi\|145597192 | A26 G34 C35 T21 | A29 G21 C20 T23 | Target Absent | A29 G20 C32 T31 |
