# Supplementary material for: Comprehensive Biothreat Cluster Identification by PCR/Electrospray-Ionization Mass Spectrometry
Source: PLoS One. 2012 Jun 29;7(6):e36528. doi: 10.1371/journal.pone.0036528 (PMC3387173; doi:10.1371/journal.pone.0036528)
Supplement: Table S5 — Experimental data on Yersinia pestis from the USAMRIID Collection. (DOCX) [file pone.0036528.s009.docx]

Table S5. Experimental data on *Yersinia pestis* from the USAMRIID Collection

| **Organism** | **Product #** | **"Old Number"** | **"Common Name"** | **Expected phenotype** | **YP/EC/Shigella (BCT358)** | **YP_INV (BCT2326)** | **YP_PLA (BCT2337)** | **YP_CAF (BCT2339)** |
| --- | --- | --- | --- | --- | --- | --- | --- | --- |
| Y. pestis (Antigua; PGM+) | YERS016-BATT02 | YE0232 | Antigua; PGM+ | PLA+/CAF+ | A26 G34 C35 T21 | A29 G21 C20 T23 | A21 G16 C18 T24 | A29 G20 C32 T31 |
| Y. pestis (CO92;PW) | YERS023-BZ023 | YE0867 | CO92;PW | PLA+/CAF+ | A26 G34 C35 T21 | A29 G21 C20 T23 | A21 G16 C18 T24 | A29 G20 C32 T31 |
| Y. pestis (PBM19:PGM+) | YERS018-BATT02 | YE0192 | PBM19:PGM+ | PLA+/CAF+ | A26 G34 C35 T21 | A29 G21 C20 T23 | A21 G16 C18 T24 | A29 G20 C32 T31 |
| Y. pestis (Pestoides B) | YERS019-BATT02 | YE0207 | Pestoides B | PLA+/CAF+ | A26 G34 C35 T21 | A29 G21 C20 T23 | A21 G16 C18 T24 | A29 G20 C32 T31 |
| Y. pestis (Nairobi) | YERS017-BATT02 | YE0237 | Nairobi | PLA+/CAF- | A26 G34 C35 T21 | A29 G21 C20 T23 | A21 G16 C18 T24 | Target Absent |
| Y. pestis Java 9 | YERS022-BATT02 | YE0533 | Java 9 | PLA+/CAF- | A26 G34 C35 T21 | A29 G21 C20 T23 | A21 G16 C18 T24 | Target Absent |
| Y. pestis (Pestoides F) | YERS020-BATT02 | YE0211 | Pestoides F | PLA-/CAF+ | A26 G34 C35 T21 | A29 G21 C20 T23 | Target Absent | A29 G20 C32 T31 |
