# Supplementary material for: Comprehensive Biothreat Cluster Identification by PCR/Electrospray-Ionization Mass Spectrometry
Source: PLoS One. 2012 Jun 29;7(6):e36528. doi: 10.1371/journal.pone.0036528 (PMC3387173; doi:10.1371/journal.pone.0036528)
Supplement: Table S6 — Experimental data on Yersinia pestis near neighbors. (DOCX) [file pone.0036528.s010.docx]

Table S6. Experimental data on *Yersinia pestis* near neighbors

| **Organism** | **Strain** | **VALS_(BCT358)** | **YP_INV (BCT2326)** | **YP_PLA (BCT2337)** | **YP_CAF (BCT2339)** |
| --- | --- | --- | --- | --- | --- |
| *Y. frederiksenii* | ATCC29912 | A24 G32 C34 T26 | Target Absent | Target Absent | Target Absent |
| *Y. frederiksenii* | ATCC33641 | A27 G32 C34 T23 | Target Absent | Target Absent | Target Absent |
| *Y. frederiksenii* | ATCC33644 | A28 G35 C31 T22 | Target Absent | Target Absent | Target Absent |
| *Y. enterocolitica* | 8081 | A26 G34 C31 T25 | Target Absent | Target Absent | Target Absent |
| *Y. enterocolitica* | DATR | A25 G34 C30 T27 | Target Absent | Target Absent | Target Absent |
| *Y. enterocolitica* | E265 | A25 G31 C35 T25 | Target Absent | Target Absent | Target Absent |
| *Y. enterocolitica* | subsp. enterocolitica ATCC23715 | A26 G34 C31 T25 | Target Absent | Target Absent | Target Absent |
| *Y. kristensenii* | ATCC33638 | A26 G35 C30 T25 | Target Absent | Target Absent | Target Absent |
| *Y. kristensenii* | ATCC33639 | A25 G36 C30 T25 | Target Absent | Target Absent | Target Absent |
| *Y. pseudotuberculosis* | ATCC11960 | A25 G35 C34 T22 | Target Absent | Target Absent | Target Absent |
| *Y. pseudotuberculosis* | IP 31758 | A25 G35 C34 T22 | Target Absent | Target Absent | Target Absent |
| *Y. pseudotuberculosis* | IP 32953 | A25 G35 C34 T22 | Target Absent | Target Absent | Target Absent |
| *Y. rohdei* | ATCC43873 | A25 G35 C32 T24 | Target Absent | Target Absent | Target Absent |
| *Y. ruckeri* | ATCC29473 | A25 G38 C31 T22 | Target Absent | Target Absent | Target Absent |
| *Y. ruckeri* | ATCC29908 | A26 G30 C34 T26 | Target Absent | Target Absent | Target Absent |
