# Supplementary material for: Comprehensive Biothreat Cluster Identification by PCR/Electrospray-Ionization Mass Spectrometry
Source: PLoS One. 2012 Jun 29;7(6):e36528. doi: 10.1371/journal.pone.0036528 (PMC3387173; doi:10.1371/journal.pone.0036528)
Supplement: Table S7 — Francisella tularensis signatures from genome sequence data and experimental measurements. (DOCX) [file pone.0036528.s011.docx]

Table S7. *Francisella tularensis* signatures from genome sequence data and experimental measurements

| **Organism** | **Strain** | | **Type** | **Source** | **FT_ASD (BCT2328)** | | | **FT_GALE (BCT2332)** | | **Data Source** | |  |  |
| --- | --- | --- | --- | --- | --- | --- | --- | --- | --- | --- | --- | --- | --- |
| *Francisella tularensis* | | subsp. tularensis, strain: FSC198 | | | | A.I | gi\|110669657 | | A16 G24 C10 T32 | | A31 G21 C13 T36 | | GenBank/ Complete Genome |
| *Francisella tularensis* | | subsp. tularensis str. NE061598 | | | | A.I | gi\|282158286 | | A16 G24 C10 T32 | | A31 G21 C13 T36 | |  |
| *Francisella tularensis* | | subsp. tularensis str. Schu S4 | | | | A.I | gi\|255961454 | | A16 G24 C10 T32 | | A31 G21 C13 T36 | |  |
| *Francisella tularensis* | | subsp. tularensis, strain: WY96-3418 | | | | A.II | gi\|134301169 | | A16 G24 C10 T32 | | **A31 G21 C14 T35** | |  |
| *Francisella tularensis* | | subsp. mediaasiatica str. FSC147 | | | | Mediaasiatica | gi\|187930913 | | A16 G24 C10 T32 | | **A31 G21 C14 T35** | |  |
| *Francisella tularensis* | | subsp. novicida, strain: U112 | | | | Novicida | gi\|118496615 | | A16 G24 C10 T32 | | ***A32 G20 C14 T35*** | |  |
| *Francisella tularensis* | | subsp. tularensis (type A.I.) | | | | A.I | 32 strains | | A16 G24 C10 T32 | | A31 G21 C13 T36 | | Results of USAMRIID Francisella Collection Testing* |
| *Francisella tularensis* | | subsp. tularensis (type A.II.) | | | | A.II | 4 strains | | A16 G24 C10 T32 | | **A31 G21 C14 T35** | |  |
| *Francisella tularensis* | | subsp. holarctica | | | | B | 2 strains | | A16 G24 C10 T32 | | **A31 G21 C14 T35** | |  |
| *Francisella tularensis* | | subsp. holarctica | | | | B | 15 strains | | **A18 G22 C10 T32** | | **A31 G21 C14 T35** | |  |
| *Francisella tularensis* | | subsp. novicida | | | | Novicida | 1 strain | | A16 G24 C10 T32 | | ***A32 G20 C14 T35*** | |  |
| *Francisella philomiragia* | | subsp. philomiragia | | | | Philomiragia | 3 strains | | ***A16 G24 C08 T34*** | | ***A32 G24 C14 T31*** | |  |
