# Supplementary material for: Comprehensive Biothreat Cluster Identification by PCR/Electrospray-Ionization Mass Spectrometry
Source: PLoS One. 2012 Jun 29;7(6):e36528. doi: 10.1371/journal.pone.0036528 (PMC3387173; doi:10.1371/journal.pone.0036528)
Supplement: Table S8 — Expected Vibrio species signatures. (DOCX) [file pone.0036528.s012.docx]

Table S8. Expected *Vibrio* species signatures^1^

| **Species** | **Strain** | **BCT2927** | **BCT2323** | **BCT2012** |
| --- | --- | --- | --- | --- |
| *Vibrio cholerae* | RC 104;CO-393;NT-642; RC 33;MO-045; EM-0363;O139; EM-0206; biovar eltor; serovar O:1; serotype Inaba;N16961;A | A35 G22 C24 T24 | A31 G23 C16 T23 | A28 G27 C22 T19 |
| *Vibrio cholerae* | RC 125;B-01; RC 776;O395; O1 classical; Pacini; serotype Hikojima;ATCC11558; serovar O:1; serotype Inaba; NCTC 7254;ATCC14034; EB-0185;O1 Inaba ElTor; serotype O1; Biotype El Tor strain N16961 | A35 G21 C24 T25 | A31 G23 C16 T23 | A28 G27 C22 T19 |
| *Vibrio alginolyticus* | ATCC14582 | A34 G19 C25 T27 |  |  |
| *Vibrio vulnificus* | ATCC33148 | A32 G21 C29 T23 |  |  |
| *Vibrio vulnificus* | CMCP6, YJ016, ATCC 27562, CIP-16, CIP 75-4, RC260 | A33 G20 C29 T23 |  |  |
| *Vibrio fischeri* | ES114, WH1, SI1D, SA1, SI6 | A32 G19 C21 T33 |  |  |
| *Vibrio fischeri* | EB12, ESP915, EM17, CG101, ET101, ET301, ET401 | A32 G19 C23 T31 |  |  |
| *Vibrio fischeri* | SR5 | A33 G19 C23 T30 |  |  |
| *Vibrio parahaemolyticus* | RIMD 2210633 | A33 G20 C24 T28 |  |  |
| *Vibrio parahaemolyticus* | ATCC17802 | A33 G20 C25 T27 |  |  |
| *Vibrio parahaemolyticus* | ATCC27519 | A34 G20 C24 T27 |  |  |
| *Vibrio parahaemolyticus* | RC 304; ATCC17803, CIP-18,UM4233, CIP 75-2 | A33 G20 C25 T27 |  |  |
| *Vibrio harveyi* | ATCCBAA-1116 | A33 G20 C25 T27 |  |  |
| *Vibrio sp.* | ATCC11985 | A33 G20 C25 T27 |  |  |
| *Vibrio mimicus* | RC 217;MB-375 | A32 G19 C27 T27 |  |  |
| *Vibrio mimicus* | RC 219;MB-451 | A31 G20 C27 T27 |  |  |
| *Vibrio mimicus* | RC 57;UM4208 | A32 G20 C26 T27 |  |  |
| *Vibrio mimicus* | RC 43, RC57 | A31 G21 C27 T26 |  |  |
| *Vibrio metschnikovii* | ATCC700040 | A33 G19 C27 T26 |  |  |
| *Vibrio natriegens* | ATCC14048 | A35 G22 C24 T24 |  |  |
| *Vibrio proteolyticus* | ATCC15338 | A35 G22 C24 T24 |  |  |
| *Vibrio logei* | SR181 | A32 G19 C23 T31 |  |  |

^1^ Multiple strains with identical signatures are captured in a single line.
