# Supplementary material for: Comprehensive Biothreat Cluster Identification by PCR/Electrospray-Ionization Mass Spectrometry
Source: PLoS One. 2012 Jun 29;7(6):e36528. doi: 10.1371/journal.pone.0036528 (PMC3387173; doi:10.1371/journal.pone.0036528)
Supplement: Table S9 — Expected Burkholderia species signatures. (DOCX) [file pone.0036528.s013.docx]

Table S9. Expected *Burkholderia* species signatures^1^

| **Burkholderia species and strain(s)** | **BCT1070** | **BCT1071** |
| --- | --- | --- |
| *B. mallei* ATCC 23344, SAVP1, NCTC 10229, NCTC 10247 | *A17 G42 C30 T18* | *A11 G25 C23 T13* |
| *B. pseudomallei 1106a,* 1710b |  |  |
| *B. pseudomallei 668, K96243,* MSHR346 | *A18 G41 C30 T18* | *A12 G24 C23 T13* |
| *B. xenovorans* LB400 |  |  |
| *B. thailandensis* E264 | A20 G41 C30 T16 | A14 G24 C23 T11 |
| *B. phymatum* STM815 | A17 G43 C28 T19 | A11 G26 C21 T14 |
| *B. phytofirmans* PsJN | A18 G40 C28 T21 | A12 G23 C22 T15 |
| *B. glumae* BGR1 | A20 G39 C30 T18 | A13 G23 C23 T13 |
| *B. sp.* CCGE1002 | A19 G40 C29 T19 | A13 G23 C23 T13 |
| *B. sp.* 383 | A18 G40 C28 T19 | A12 G23 C21 T14 |
| *B. cenocepacia* AU 1054, HI2424, MCO-3 | A19 G39 C28 T19 | A12 G23 C21 T14 |
| *B. cenocepacia* J2315 | A18 G40 C27 T20 | A11 G24 C20 T15 |
| *B. multivorans* ATCC 17616 | A18 G41 C28 T18 | A12 G24 C21 T13 |
| *B. vietnamiensis* G4 | A17 G42 C29 T17 | A11 G25 C22 T12 |
| *B. ambifaria* AMMD, MC40-6 | A18 G40 C29 T18 | A12 G23 C22 T13 |

^1^ Multiple strains with identical signatures are captured in a single line.
