# Supplementary material for: Comprehensive Biothreat Cluster Identification by PCR/Electrospray-Ionization Mass Spectrometry
Source: PLoS One. 2012 Jun 29;7(6):e36528. doi: 10.1371/journal.pone.0036528 (PMC3387173; doi:10.1371/journal.pone.0036528)
Supplement: Table S10 — Expected Brucella species signatures. (DOCX) [file pone.0036528.s014.docx]

Table S10. Expected *Brucella* species signatures

| **Organism** | **Source** | **BCT_1111** | **BCT_1112** |  |
| --- | --- | --- | --- | --- |
| *Brucella abortus* | gi\|189018840 | A29 G37 C23 T15 | A23 G37 C27 T14 | GenBank Data |
| *Brucella abortus* | gi\|189023268 | A29 G37 C23 T15 | A23 G37 C27 T14 |  |
| *Brucella abortus* | gi\|62195123 | A29 G37 C23 T15 | A23 G37 C27 T14 |  |
| *Brucella abortus* | gi\|62288991 | A29 G37 C23 T15 | A23 G37 C27 T14 |  |
| *Brucella abortus* | gi\|82615033 | A29 G37 C23 T15 | A23 G37 C27 T14 |  |
| *Brucella abortus* | gi\|82698932 | A29 G37 C23 T15 | A23 G37 C27 T14 |  |
| *Brucella canis* | gi\|161334802 | A29 G37 C23 T15 | A23 G37 C27 T14 |  |
| *Brucella canis* | gi\|161617991 | A29 G37 C23 T15 | A23 G37 C27 T14 |  |
| *Brucella melitensis* | gi\|17986242 | A29 G37 C23 T15 | A23 G37 C27 T14 |  |
| *Brucella melitensis* | gi\|17986284 | A29 G37 C23 T15 | A23 G37 C27 T14 |  |
| *Brucella melitensis* | gi\|225639911 | A29 G37 C23 T15 | A23 G37 C27 T14 |  |
| *Brucella melitensis* | gi\|225851546 | A29 G37 C23 T15 | A23 G37 C27 T14 |  |
| *Brucella melitensis* | gi\|82698932 | A29 G37 C23 T15 | A23 G37 C27 T14 |  |
| *Brucella microti* | gi\|255998623 | A29 G37 C23 T15 | A23 G37 C27 T14 |  |
| *Brucella microti* | gi\|256368465 | A29 G37 C23 T15 | A23 G37 C27 T14 |  |
| *Brucella ovis* | gi\|148370077 | A29 G37 C23 T15 | A23 G37 C27 T14 |  |
| *Brucella ovis* | gi\|148558820 | A29 G37 C23 T15 | A23 G37 C27 T14 |  |
| *Brucella suis* | gi\|163673000 | A29 G37 C23 T15 | A23 G37 C27 T14 |  |
| *Brucella suis* | gi\|163842277 | A29 G37 C23 T15 | A23 G37 C27 T14 |  |
| *Brucella suis* | gi\|23500916 | A29 G37 C23 T15 | A23 G37 C27 T14 |  |
| *Brucella suis* | gi\|54112365 | A29 G37 C23 T15 | A23 G37 C27 T14 |  |
| *Brucella suis* | gi\|56968325 | A29 G37 C23 T15 | A23 G37 C27 T14 |  |
| *Brucella abortus* | BRUC012-AFIP01 | A29 G37 C23 T15 | A23 G37 C27 T14 | USAMRIID Samples |
| *Brucella canis* | BRUC014-AFIP01 | A29 G37 C23 T15 | A23 G37 C27 T14 |  |
| *Brucella maris* | BRUC015-AFIP01 | A29 G37 C23 T15 | A23 G37 C27 T14 |  |
| *Brucella melitensis* | BRUC013-AFIP01 | A29 G37 C23 T15 | A23 G37 C27 T14 |  |
| *Brucella neotomae* | BRUC017-AFIP01 | A29 G37 C23 T15 | A23 G37 C27 T14 |  |
| *Brucella ovis* | BRUC018-AFIP01 | A29 G37 C23 T15 | A23 G37 C27 T14 |  |
| *Brucella suis* | BRUC016-AFIP01 | A29 G37 C23 T15 | A23 G37 C27 T14 |  |
