# Supplementary material for: Comprehensive Biothreat Cluster Identification by PCR/Electrospray-Ionization Mass Spectrometry
Source: PLoS One. 2012 Jun 29;7(6):e36528. doi: 10.1371/journal.pone.0036528 (PMC3387173; doi:10.1371/journal.pone.0036528)
Supplement: Table S11 — Expected Clostridium species signatures. (DOCX) [file pone.0036528.s015.docx]

Table S11. Expected *Clostridium* species signatures

| **Organism** | **Strain** | **Clostridium (BCT1075)** | **Clostridium (BCT1076)** | **PLEX-ID Clusters** |
| --- | --- | --- | --- | --- |
| *Clostridium botulinum A* | ATCC19397 | A22 G23 C9 T14 | A22 G21 C8 T13 | 1 |
| *Clostridium botulinum A* | ATCC3502 | A22 G23 C9 T14 | A22 G21 C8 T13 |  |
| *Clostridium botulinum A* | Hall | A22 G23 C9 T14 | A22 G21 C8 T13 |  |
| *Clostridium botulinum B1* | Okra | A22 G23 C9 T14 | A22 G21 C8 T13 |  |
| *Clostridium botulinum F* | 230613 | A22 G23 C9 T14 | A22 G21 C8 T13 |  |
| *Clostridium botulinum F* | Langeland | A22 G23 C9 T14 | A22 G21 C8 T13 |  |
| *Clostridium botulinum* | JHUCB001 | A23 G22 C9 T14 | A23 G20 C8 T13 | 2 |
| *Clostridium botulinum A2* | Kyoto | A23 G22 C9 T14 | A23 G20 C8 T13 |  |
| *Clostridium botulinum A3* | Loch Maree | A23 G22 C9 T14 | A23 G20 C8 T13 |  |
| *Clostridium botulinum Ba4* | 657 | A23 G22 C9 T14 | A23 G20 C8 T13 |  |
| *Clostridium sporogenes* | NoStrain_101_0 | A23 G22 C9 T14 | A23 G20 C8 T13 |  |
| *Clostridium botulinum B* | Eklund 17B | A24 G22 C9 T15 | A24 G20 C8 T14 | 3 |
| *Clostridium botulinum E3* | Alaska E43 | A24 G22 C9 T15 | A24 G20 C8 T14 |  |
| *Clostridium ljungdahlii* | DSM 13528 | A24 G22 C9 T15 | A24 G20 C8 T14 |  |
| *Clostridium perfringens* | 13 | A24 G22 C9 T15 | A24 G20 C8 T14 |  |
| *Clostridium perfringens* | ATCC13124 | A24 G22 C9 T15 | A24 G20 C8 T14 |  |
| *Clostridium perfringens* | SM101 | A24 G22 C10 T14 | A24 G20 C9 T13 | 4 |
| *Clostridium tetani* | 88 | A25 G25 C10 T17 | A25 G23 C9 T16 | 5 |
| *Clostridium tetani* | Massachusetts | A25 G25 C10 T17 | A25 G23 C9 T16 |  |
| *Clostridium difficile* | 630 | A22 G22 C9 T13 | A22 G20 C8 T12 | 6 |
| *Clostridium difficile* | 2007855 | A22 G22 C9 T13 | A22 G20 C8 T12 |  |
| *Clostridium difficile* | BI1 | A22 G22 C9 T13 | A22 G20 C8 T12 | 7 |
| *Clostridium difficile* | BI9 | A22 G22 C9 T13 | A22 G20 C8 T12 |  |
| *Clostridium difficile* | CD196 | A22 G22 C9 T13 | A22 G20 C8 T12 |  |
| *Clostridium difficile* | CF5 | A22 G22 C9 T13 | A22 G20 C8 T12 |  |
| *Clostridium difficile* | M68 | A22 G22 C9 T13 | A22 G20 C8 T12 |  |
| *Clostridium difficile* | R20291 | A22 G22 C9 T13 | A22 G20 C8 T12 |  |
| *Clostridium difficile* | M120 | A23 G22 C9 T12 | A23 G20 C8 T11 | 8 |
| *Clostridium acetobutylicum* | ATCC 824D | A24 G21 C8 T15 | A24 G19 C7 T14 | 9 |
| *Clostridium acetobutylicum* | ATCC824 | A24 G21 C8 T15 | A24 G19 C7 T14 |  |
| *Clostridium beijerinckii* | NCIMB 8052 | Does Not Amplify | A24 G20 C8 T14 |  |
| *Clostridium cellulolyticum* | H10 | A25 G24 C10 T19 | A25 G22 C9 T18 |  |
| *Clostridium cellulovorans* | 743B | A28 G22 C8 T20 | A28 G20 C7 T19 |  |
| *Clostridium novyi* | NT | A24 G23 C9 T17 | A24 G21 C8 T16 |  |
| *Clostridium saccharolyticum* | WM1 | A46 G51 C31 T35 | A46 G49 C30 T34 |  |
| *Clostridium thermocellum* | ATCC27405 | A27 G33 C14 T23 | A27 G31 C13 T22 |  |
