# Supplementary material for: Comprehensive Biothreat Cluster Identification by PCR/Electrospray-Ionization Mass Spectrometry
Source: PLoS One. 2012 Jun 29;7(6):e36528. doi: 10.1371/journal.pone.0036528 (PMC3387173; doi:10.1371/journal.pone.0036528)
Supplement: Table S12 — Expected Coxiella species signatures. (DOCX) [file pone.0036528.s016.docx]

Table S12. Expected *Coxiella* species signatures

| **Organism** | **Strain** | **Coxiella (BCT1079)** | **Coxiella (BCT1080)** |
| --- | --- | --- | --- |
| *Coxiella burnetii* | CbuG_Q212 | A26 G21 C13 T12 | A19 G31 C16 T23; A19 G31 C17 T22 |
| *Coxiella burnetii* | CbuK_Q154 | A26 G21 C13 T12 | A19 G31 C16 T23 |
| *Coxiella burnetii* | Dugway 7E9-12 | A26 G21 C13 T12 | A19 G31 C16 T23 |
| *Coxiella burnetii* | Nine Mile (RSA 493) | A26 G21 C13 T12 | A19 G31 C16 T23 |
| *Coxiella burnetii* | RSA 331 | A26 G21 C13 T12 | A19 G31 C16 T23 |
| *Coxiella burnetii* | RSA 493 | A26 G21 C13 T12 | A19 G31 C16 T23 |
