# Supplementary material for: Comprehensive Biothreat Cluster Identification by PCR/Electrospray-Ionization Mass Spectrometry
Source: PLoS One. 2012 Jun 29;7(6):e36528. doi: 10.1371/journal.pone.0036528 (PMC3387173; doi:10.1371/journal.pone.0036528)
Supplement: Table S13 — Expected Rickettsia species signatures. (DOCX) [file pone.0036528.s017.docx]

Table S13. Expected *Rickettsia* species signatures and clusters based on biothreat assay data

| **Organism** | **Strain** | **Rickettsia (BCT1083)** | **Rickettsia (BCT1084)** | **PLEX-ID Clusters** |
| --- | --- | --- | --- | --- |
| *Rickettsia prowazekii* | Madrid E | A46 G32 C27 T39 | A32 G20 C17 T31 | 1 |
| *Rickettsia prowazekii* | Rp22 | A46 G32 C27 T39 | A32 G20 C17 T31 |  |
| *Rickettsia prowazekii* | TIGER0411 | A46 G32 C27 T39 | A32 G20 C17 T31 |  |
| *Rickettsia prowazekii* | TIGER0422 | A46 G32 C27 T39 | A32 G20 C17 T31 |  |
| *Rickettsia typhi* | TIGER0409 | A47 G31 C29 T37 | A33 G19 C18 T30 | 2 |
| *Rickettsia typhi* | TIGER0410 | A47 G31 C29 T37 | A33 G19 C18 T30 |  |
| *Rickettsia typhi* | Wilmington | A47 G31 C29 T37 | A33 G19 C18 T30 |  |
| *Rickettsia rickettsii* | Iowa | A40 G34 C30 T31 | A25 G22 C21 T23 | 3 |
| *Rickettsia rickettsii* | NC_RMSF 3 | A40 G34 C30 T31 | A25 G22 C21 T23 |  |
| *Rickettsia rickettsii* | Sheila Smith | A40 G34 C30 T31 | A25 G22 C21 T23 |  |
| *Rickettsia rickettsii* | TIGER0412 | A40 G34 C30 T31 | A25 G22 C21 T23 |  |
| *Rickettsia africae* | ESF-5 | A43 G31 C30 T31 | A27 G20 C21 T23 | 4 |
| *Rickettsia africae* | TIGER0416 | A43 G31 C30 T31 | A27 G20 C21 T23 |  |
| *Rickettsia akari* | Hartford | A43 G31 C30 T31 | A27 G20 C21 T23 |  |
| *Rickettsia amblyommii* | CDC-000027 | A41 G33 C30 T31 | A25 G22 C21 T23 | 5 |
| *Rickettsia australis* | TIGER0413 | A41 G33 C30 T31 | A25 G22 C21 T23 |  |
| *Rickettsia conorii* | Malish 7 | A41 G33 C30 T31 | A25 G22 C21 T23 | 7 |
| *Rickettsia conorii* | TIGER0408 | A41 G33 C30 T31 | A25 G22 C21 T23 |  |
| *Rickettsia felis* | URRWXCal2 | A41 G33 C30 T31 | A25 G22 C21 T23 |  |
| *Rickettsia felis* | URRWXCal2; California 2 | A41 G33 C30 T31 | A25 G22 C21 T23 |  |
| *Rickettsia helvetica* | NOLTE-00003 | A41 G33 C30 T31 | A25 G22 C21 T23 |  |
| *Rickettsia massiliae* | MTU5 | A41 G33 C30 T31 | A25 G22 C21 T23 |  |
| *Rickettsia monacensis* | GER_141 | A42 G32 C30 T31 | A26 G21 C21 T23 | 8 |
| *Rickettsia parkeri* | TIGER0415 | A42 G32 C30 T31 | A26 G21 C21 T23 |  |
| *Rickettsia sibirica* | TIGER0414 | A43 G32 C30 T31 | A27 G21 C21 T23 | 9 |
| *Rickettsia sibirica* | Zdrodovskii | A43 G32 C30 T31 | A27 G21 C21 T23 |  |
| *Rickettsia peacockii* | Rustic | A41 G33 C30 T31 | A25 G22 C21 T23 | 10 |
| *Rickettsia bellii* | OSU 85-389 | A42 G33 C28 T32 | A26 G22 C19 T24 | 11 |
| *Rickettsia bellii* | RML369-C | A41 G33 C28 T33 | A25 G22 C20 T24 | 12 |
| *Rickettsia canadensis* | McKiel | A42 G33 C31 T34 | A26 G22 C22 T26 | 13 |
