# Supplementary material for: Comprehensive Biothreat Cluster Identification by PCR/Electrospray-Ionization Mass Spectrometry
Source: PLoS One. 2012 Jun 29;7(6):e36528. doi: 10.1371/journal.pone.0036528 (PMC3387173; doi:10.1371/journal.pone.0036528)
Supplement: Table S14 — Expected Enterobacteriaceae species signatures. (DOCX) [file pone.0036528.s018.docx]

Table S14. Expected Enterobacteriaceae species signatures

| **Organism** | **Strain/Serotype** | **BCT358** | **PLEX-ID BC Clusters** |
| --- | --- | --- | --- |
| *Escherichia coli* | O157:H7-35150; O157:H7-43888; O157:H7-493; O157:H7-LSU-61; O157:H7-MA6; Sakai; EC4115; EDL933; O55:H7-5A | A25 G39 C29 T23 | 1 |
| *Escherichia coli* | ATCC 8739; ATCC8739; BW2952; DH10B; K-12; K-12 substr. MG1655; K-12 substr. W3110; 1712; OH0707835; SE11; UMN026 | A23 G40 C30 T23 | 2 |
| *Escherichia coli* | 536; ED1a; IAI39; 4W7005; 6W7083; 7W6863; 8W 7048; 6W 6953; 5W 7022; 8W 6899; 2W 7040; 6W6810; 6W6628; UPEC-CFT073 | A24 G39 C29 T24 | 3 |
| *Escherichia coli* | 55989; HS; IAI1; TW02918; TW07990; | A24 G39 C30 T23 | 4 |
| *Escherichia coli* | 3023-94; APEC O1; BL21; E2348/69; E24377A; 7018 6W; SW 7111; 3W 6977; MD6014; O26:H11 E coli 11368; S88; SECEC SMS-3-5; TW00016; TW00971; TW01120; TW07865; UTI89; O157:not H7-15880; | A24 G40 C29 T23 | 5 |
| *Escherichia fergusonii* | ATCC35469; |  |  |
| *Shigella dysenteriae* | Sd197; |  |  |
| *Shigella flexneri* | 301; 8401; 2457T |  |  |
| *Shigella sonnei* | Ss046; |  |  |
| *Salmonella enterica* | subsp. enterica, serovar Choleraesuis; ATCC9150; subsp. enterica BAA-1045; subsp. enterica serovar Enteritidis SARB18; subsp. enterica serovar Enteritidis str. P125109; subsp. enterica serovar Gallinarum SARB21; subsp. enterica serovar Gallinarum str. 287/91; subsp. enterica serovar Gallinarum str. ATCC 700623; subsp. enterica serovar Paratyphi A str. AKU_12601; subsp. enterica serovar Paratyphi A str. AKU_12601; subsp. enterica serovar Paratyphi A str. ATCC 9150; subsp. enterica serovar Paratyphi B str. ATCC BAA1585; subsp. enterica serovar Pullorum str. ATCC 10398; subsp. enterica serovar Pullorum str. ATCC 13036; subsp. enterica serovar Pullorum str. ATCC 19945; subsp. enterica serovar Stanley str. ATCC 7308; subsp. enterica serovar Typhi ATCC700931; subsp. enterica serovar Typhi str. CT18; subsp. enterica serovar Typhi str. Ty2; subsp. enterica serovar Worthington str. ATCC 9607 | A24 G41 C30 T21 | 6 |
| *Salmonella enterica* | subsp. enterica serovar Gaminara str. ATCC BAA711; subsp. enterica serovar Minnesota str. ATCC 49284; subsp. enterica serovar Montevideo SARB31; subsp. enterica serovar Pomona str. ATCC 10729; subsp. enterica serovar Sandiego str. ATCC 23199; subsp. enterica serovar Senftenberg str. ATCC 8400; subsp. enterica serovar Urbana str. ATCC 9261 | A24 G42 C29 T21 | 7 |
| *Salmonella enterica* | subsp. arizonae serovar Arizonae ATCC13314; subsp. enterica serovar Adelaide str. ATCC 10718; subsp. enterica serovar Chester str. ATCC 11997; subsp. enterica serovar Rissen 510 | A25 G40 C30 T21 | 8 |
| *Salmonella enterica* | subsp. enterica serovar Abortusovis ATCC6952; Type I; subsp. enterica serovar Anatum str. ATCC 9270; subsp. enterica serovar Anatum str. ATCC BAA1592; subsp. enterica serovar Enteritidis SARB19; subsp. enterica serovar Hadar str. ATCC 51956; subsp. enterica serovar Inverness str. ATCC 10720; subsp. enterica serovar Newport SARB37; subsp. enterica serovar Newport SARB38; subsp. enterica serovar Newport str. SL254; subsp. enterica serovar Typhimurium SARB68; subsp. enterica serovar Typhimurium str. LT2 | A22 G42 C30 T22 | 9 |
| *Salmonella enterica* | subsp. enterica serovar Cerro str. ATCC 10723; subsp. enterica serovar Indiana str. ATCC 51959; subsp. enterica serovar Kentucky str. ATCC 9263; subsp. enterica serovar Mbandaka str. ATCC 51958; subsp. enterica serovar Paratyphi B str. ATCC 51962; subsp. enterica serovar Virchow str. ATCC 51955 | A22 G42 C31 T21 | 10 |
| *Salmonella enterica* | subsp. enterica serovar Agona str. ATCC 51957; subsp. enterica serovar Agona str. ATCC BAA707; subsp. enterica serovar Agona str. SL483; subsp. enterica serovar Berta str. ATCC 8392; subsp. enterica serovar Senftenberg SARB59 | A23 G41 C30 T22 | 11 |
| *Salmonella enterica* | subsp. enterica serovar Bredeney str. ATCC 10728; subsp. enterica serovar Panama str. ATCC 7378; subsp. enterica serovar Reading str. ATCC 6967; subsp. enterica serovar Schwarzengrund str. CVM19633 | A23 G41 C31 T21 | 12 |
| *Salmonella houtenae* | Type IV |  |  |
| *Salmonella Type VII* | Type VII |  |  |
| *Escherichia vulneris* | ATCC33832 |  |  |
| *Salmonella enterica* | subsp. enterica serovar Albany str. ATCC 51960; subsp. enterica serovar Bareilly str. ATCC 9115; subsp. enterica serovar Blockley str. ATCC 51961; subsp. enterica serovar Derby str. ATCC 6960; subsp. enterica serovar Paratyphi B SARB43; subsp. enterica serovar Paratyphi B str. SPB7 | A23 G42 C30 T21 | 13 |
| *Salmonella enterica* | subsp. enterica serovar Dublin str. ATCC 15480; subsp. enterica serovar Dublin str. ATCC 39184; subsp. enterica serovar Dublin str. ATCC BAA1514; subsp. enterica serovar Dublin str. CT_02021853; subsp. enterica serovar Saintpaul SARB55; subsp. enterica serovar Saintpaul SARB56 | A23 G43 C28 T22 | 14 |
| *Salmonella enterica* | subsp. enterica serovar Enterica PA tomato 2004 outbreak str. ATCC BAA1604; subsp. enterica serovar Thompson SARB62; subsp. enterica serovar Thompson str. ATCC 8391 | A24 G38 C32 T22 | 15 |
| *Salmonella enterica* | subsp. enterica BAA-1587; subsp. enterica serovar Choleraesuis str. SC-B67; subsp. enterica serovar Enteritidis SARB17; subsp. enterica serovar Give str. ATCC 9268; subsp. enterica serovar Heidelberg SARB23; subsp. enterica serovar Heidelberg str. SL476; subsp. enterica serovar Infantis str. ATCC 51741; subsp. enterica serovar Paratyphi C str. RKS4594; subsp. enterica serovar Poona str. ATCC BAA1673 | A24 G40 C31 T21 | 16 |
| *Salmonella salamae* | Type II | A24 G40 C32 T20 | 17 |
| *Salmonella diarizonae* | Type IIIB | A22 G40 C32 T22 | 18 |
| *Salmonella indica* | Type VI | A24 G42 C30 T20 | 19 |
| *Salmonella enterica* | subsp. enterica serovar Newport SARB36 | A22 G43 C30 T21 | 20 |
| *Salmonella enterica* | subsp. arizonae serovar Arizonae | A26 G39 C30 T21 | 21 |
| *Salmonella enterica* | subsp. enterica serovar Paratyphi B str. ATCC 8759 | A22 G41 C32 T21 | 22 |
| *Salmonella enterica* | subsp. enterica serovar Rubislaw str. ATCC 10717 | A24 G42 C28 T22 | 23 |
| *Escherichia coli* | ATCC4157 | A24 G40 C30 T22 | 24 |
| *Shigella boydii* | Sb227 | A22 G41 C30 T23 | 25 |
| *Escherichia vulneris* | ATCC39368 | A22 G41 C31 T22 | 26 |
| *Escherichia vulneris* | ATCC29943 | A23 G40 C31 T22 | 27 |
| *Escherichia fergusonii* | ATCC35473 | A23 G41 C30 T22 | 28 |
| *Escherichia hermannii* | ATCC33650 | A24 G41 C29 T22 | 29 |
