# Supplementary material for: Comprehensive Biothreat Cluster Identification by PCR/Electrospray-Ionization Mass Spectrometry
Source: PLoS One. 2012 Jun 29;7(6):e36528. doi: 10.1371/journal.pone.0036528 (PMC3387173; doi:10.1371/journal.pone.0036528)
Supplement: Table S15 — Expected Shigella species signatures. (DOCX) [file pone.0036528.s019.docx]

Table S15. Expected Shigella species signatures

| **Organism** | **Strain** | **Shigella (BCT1105)** | **Shigella (BCT1106)** |
| --- | --- | --- | --- |
| *Shigella boydii* | Sb227 | A19 G18 C21 T12 | A16 G18 C26 T19 |
| *Shigella dysenteriae* | Sd197 | A19 G18 C21 T12 | A16 G18 C26 T19 |
| *Shigella flexneri* | 301 | A19 G18 C21 T12 | A16 G18 C26 T19 |
| *Shigella flexneri* | 8401 | A19 G18 C21 T12 | A16 G18 C26 T19 |
| *Shigella flexneri* | 2457T | A19 G18 C21 T12 | A16 G18 C26 T19 |
| *Shigella sonnei* | Ss046 | A19 G18 C21 T12 | A16 G18 C26 T19 |
