# Supplementary material for: Comprehensive Biothreat Cluster Identification by PCR/Electrospray-Ionization Mass Spectrometry
Source: PLoS One. 2012 Jun 29;7(6):e36528. doi: 10.1371/journal.pone.0036528 (PMC3387173; doi:10.1371/journal.pone.0036528)
Supplement: Table S16 — Expected Alphavirus signatures. (DOCX) [file pone.0036528.s020.docx]

Table S16. Expected Alphavirus signatures

| **Organism** | **Strain** | **Alphavirus (VIR966)** | **Alphavirus (VIR2499)** | **PLEX-ID Cluster** |
| --- | --- | --- | --- | --- |
| Eastern equine encephalitis virus | BeAr436087 | A26 G23 C23 T26 | A24 G29 C25 T22 I5 | 1 |
|  | PE-3.0815 | A26 G24 C27 T21 | A24 G29 C25 T22 I5 | 2 |
|  | FL93-939; Florida91-4697; Georgia 97; Georgia 97 (North America, 1997); NJ/60; North American; PE6 | A26 G27 C22 T23 | A23 G31 C28 T18 I5 | 3 |
|  | PE6 | A26 G27 C22 T23 | A24 G30 C28 T18 I5 | 4 |
|  | PE-0.0155 | A27 G25 C25 T21 | A24 G29 C26 T21 I5 | 5 |
| Highlands J virus | 585-01; 744-01; B-230 | A26 G28 C22 T22 | A28 G29 C27 T16 I5 | 6 |
| Venezuelan equine encephalitis virus | Everglades Fe3-7c; Everglades virus | A24 G26 C25 T23 | A27 G28 C25 T20 I5 | 7 |
|  | 78V3531 | A25 G24 C24 T25 | A25 G29 C26 T20 I5 | 8 |
|  | Tonate CaAn 410d | A25 G25 C25 T23 | A26 G30 C27 T17 I5 | 9 |
|  | Cabassou CaAr 508 | A25 G25 C25 T23 | A27 G30 C25 T18 I5 | 10 |
|  | 8131 | A25 G25 C25 T23 | A29 G27 C27 T17 I5 | 11 |
|  | 3908; 6119; 66457; 66637; 243937; 254934; 255010; 1D V-209-A-TVP1163; 71-180; 600035-71-180/4; 83U434; CO951006; P676; PMCHo5;SH3; TC-83; Trinidad donkey; V198; V3526; ZPC738 | A25 G25 C26 T22 | A27 G29 C27 T17 I5 | 12 |
|  | 3880 | A25 G25 C26 T22 | A29 G27 C27 T17 I5 | 13 |
|  | 68U201; 80U76; CPA152; CPA201; MX01-22; OAX131; OAX142 | A25 G27 C22 T24 | A27 G29 C26 T18 I5 | 14 |
|  | Pixuna BeAr 35645 | A25 G27 C23 T23 | A28 G26 C28 T18 I5 | 15 |
|  | AG80-663 | A26 G22 C25 T25 | A27 G26 C23 T24 I5 | 16 |
|  | 71D1252 | A26 G25 C24 T23 | A26 G27 C28 T19 I5 | 17 |
|  | Mena II | A26 G26 C22 T24 | A27 G29 C26 T18 I5 | 18 |
|  | Mucambo BeAn 8 | A27 G23 C26 T22 | A27 G28 C26 T19 I5 | 19 |
| Western equine encephalomyelitis virus | CO92-1356 | A26 G27 C23 T22 | A27 G29 C23 T21 I5 | 20 |
|  | 71V1658; 71V1658(OR71); 85-452NM; BFS-2005; Imperial; Kern; Montana-64; TBT 235 | A26 G27 C23 T22 | A28 G28 C23 T21 I5 | 21 |
|  | McMillan | A26 G28 C22 T22 | A28 G28 C22 T22 I5 | 22 |
| Aura virus | BeAR10315 | A25 G22 C26 T25 |  | 23 |
| Barmah Forest virus | BH2193 | A30 G23 C23 T22 |  | 24 |
| Chikungunya virus | IPD/A SH 2807 | A29 G21 C24 T24 |  | 25 |
|  | SGEHICHD13508; SGEHICHS277108 | A29 G21 C25 T23 |  | 26 |
|  | 37997; A301; ALSA-1; Angola M2022; AR 18211; ArA 2657; ArA 30548; ArD 30237; ArD 93229; CAR256; DakAr B 16878; HB78;HD 180760; IbAn4824; IbH35; IND-00-MH4; PM2951; Ross; Ross low-psg; S27; SAH2123; SH 3013; SH2830; UgAg4155; Vereeniging | A30 G20 C24 T24 |  | 27 |
|  | 05-061;05-115;05-209; 06-021;06-027;06-049; 0611aTw;0706aTw; 0810aTw; 0810bTw; 1455-75; 3412-78; 6441-88; AF15561; BNI-CHIKV_899; CHIK31; CO392-95; CU-Chik_OBF; CU-Chik009; CU-Chik10; CU-Chik661; CU-Chik683; D570/06; DHS4263-Calif AB; DRDE-06; DRDEHydISW06; DRDE-07; FD080008; FD080178; FD080231; Gibbs 63-263; Hu/85/NR/001;I-634029; IND-06-AP3; IND-06-KA15; IND-06-MH2; IND-06-RJ1; IND-06-TN1; IND-63-WB1; IND-73-MH5; IND-GJ51; IND-GJ52; IND-GJ53; IND-KA51; IND-KA52; IND-KR51; IND-KR52; IND-MH51; ITA07-RA1; JKT23574; K0146-95; LK(EH)CH17708; LK(EH)CH18608; LK(EH)CH20108; LK(EH)CH4408; LK(EH)CH6708; LK(EH)CH7708; LK(EH)chik19708; LK(PB)CH1008; LK(PB)CH1608; LK(PB)CH3008;LK(PB)CH5308; LK(PB)CH5808; LK(PB)chik3408; LK(PB)chik6008; LKEHCH13908; LKMTCH2707; LKRGCH1507; LR2006_OPY1; LSFS; MY002IMR/06/BP; MY003IMR/06/BP; MY019IMR/06/BP; MY021IMR/06/BP; PhH15483; PO731460; RGCB03/KL06; RGCB05/KL06; RGCB120/KL07; RGCB355/KL08; RGCB356/KL08; RGCB80/KL07; RSU1;SD08Pan; SGEHICHD122508; SGEHICHD93508; SGEHICHD96808; SGEHICHS421708; SGEHICHS422308; SGEHICHS422808; SGEHICHS424108; SGEHICHS425208; SGEHICHT077808; SL-CK1;SL-CR 3; SL10571; SL11131; SL15649; SV0444-95; TH35; TM25; TSI-GSD-218; TSI-GSD-218-VR1; Wuerzburg | A30 G20 C25 T23 |  | 28 |
| Getah virus | HB0234; M1; swine; YN0540 | A25 G24 C25 T24 |  | 29 |
|  | LEIV 17741 MPR | A26 G23 C25 T24 |  | 30 |
|  | LEIV 16275 Mag | A26 G24 C25 T23 |  | 31 |
| Igbo Ora virus | IBH10964 | A30 G22 C24 T22 |  | 32 |
| Mayaro virus | MAYLC | A31 G22 C23 T22 |  | 33 |
|  |  | A31 G24 C22 T21 |  | 34 |
| Middelburg virus | MIDV857 | A27 G27 C22 T22 |  | 35 |
| Ndumu virus |  | A27 G26 C21 T24 |  | 36 |
| O'nyong-nyong virus | Gulu; SG650 | A31 G21 C23 T23 |  | 37 |
| Ross River virus | NB5092 | A28 G21 C26 T23 |  | 38 |
|  | 2975; 2982; 3078; 8961; 9057; DC5692; QML 1; T48 | A29 G20 C26 T23 |  | 39 |
| Sagiyama virus | Suckling mouse brain | A25 G24 C25 T24 |  | 40 |
| Semliki forest virus | strain A7 | A28 G23 C25 T22 |  | 41 |
|  | DI-19;DI-6; genome strain; L10;NoStrain_79255;original; original.1; SK prototype; ts1 mutant; ts10 mutant; ts11 mutant; ts13 mutant; ts14 mutant; ts6 mutant; ts9 mutant; pkth301 | A28 G23 C26 T21 |  | 42 |
|  | pkth301 | A28 G24 C26 T21 |  | 43 |
| Sindbis virus | MRE16 | A23 G27 C28 T20 |  | 44 |
|  | genome strain | A24 G25 C26 T23 |  | 45 |
|  | SW6562 | A25 G25 C25 T23 |  | 46 |
| Trocara virus |  | A26 G20 C24 T28 |  | 47 |
