# Supplementary material for: Comprehensive Biothreat Cluster Identification by PCR/Electrospray-Ionization Mass Spectrometry
Source: PLoS One. 2012 Jun 29;7(6):e36528. doi: 10.1371/journal.pone.0036528 (PMC3387173; doi:10.1371/journal.pone.0036528)
Supplement: Table S17 — Expected Orthopoxvirus signatures. (DOCX) [file pone.0036528.s021.docx]

Table S17. Expected *Orthopoxvirus* signatures

| **Organism** | **Strain** | **VIR979** | **VIR985** | **PLEX-ID Cluster** |
| --- | --- | --- | --- | --- |
| Variola virus | Sudan 1947 (Rumbec); Bangladesh-1975; India-1967; Afghanistan 1970 Variolator 4; Bangladesh 1974 (nur islam); Bangladesh 1974 (Shahzaman); Bangladesh 1974 (Solaiman); Bangladesh 1975 v75-550 Banu; Botswana 1972 (v72-143); Botswana 1973 (v73-225); China Horn 1948; Sabin Lab July 1948; Congo 1970 v70-46 Kinshasa; Congo 9 1970 (v74-227 Gispen); Ethiopia 1972 (Eth16 R14-1X-72 Addis); Ethiopia 1972 (Eth17 R14-1X-72 Addis); Germany 1958 Heidelberg; India 1953 (Kali-Muthu-M50 Madras); India 1953 (New Delhi); India 1964 7124 Vellore; India 1964 7125 Vellore; Iran 1972 2602 Tabriz; Japan 1946 (Yamada MS-2(A Tokyo)); Japan 1951 (Harper, Masterseed); Japan 1951 (Stillwell, Masterseed); Korea 1947 (Lee, Masterseed); Kuwait 1967 (K1629); Nepal 1973 V73-175; Pakistan 1969 (Rafig Lahore); Somalia 1977 (V77-1252); Somalia 1977 (V77-1605); Somalia 1977; V77-2479; South Africa 1965 (102 Natal, Ingwavuma); South Africa 1965 (103 T'vaal, Nelspruit); Sudan 1947 (Juba); Sumatra 1970 V70-222; Sumatra 1970 V70-228; Syria 1972 V72-199; Tanzania 1965 kembula; United Kingdom 1946 Harvey; United Kingdom 1946 Hinden (Middlesex); Yugoslavia 1972 V72-164 | A40 G24 C23 T41 | A42 G23 C24 T23 | 1 |
|  | United Kingdom 1947 Higgins (Staffordshire) | A40 G24 C23 T41 | A44 G21 C24 T23 | 2 |
|  | Benin, Dahomey 1968 (v68-59); Guinea 1969 (005); Sierra Leone 1969 (V68-258); United Kingdom 1952 Butler | A40 G24 C24 T40 | A43 G22 C24 T23 | 3 |
| Variola minor virus | Garcia-1966; Brazil 1966 (v66-39 Sao Paulo); Niger 1969 (001, importation from Nigeria) | A41 G23 C24 T40 | A43 G22 C24 T23 | 4 |
| Monkeypox virus | Congo_2003_358; COP-58;Liberia_1970_184; MPXV-WRAIR7-61; Walter Reed 267; Sierra Leone;USA_2003_039; USA_2003_044;VR-267; Zaire_1979-005; Zaire-96-I-16 | A40 G24 C25 T39 | A41 G25 C18 T28 | 5 |
| Vaccinia virus | 3737; LC16m8; LC16mO; Lister; Tian Tan | A39 G25 C27 T37 | A43 G23 C24 T22 | 6 |
|  | Acambis 3000 Modified Virus Ankara (MVA); AGR-MVA-572seq; Ankara;DUKE;MVA-572; ECACC V94012707; MVA-BN;MVA-I721; CNCM I721; Western Reserve | A40 G24 C26 T38 | A43 G23 C24 T22 | 7 |
| Camelpox virus | M-96 | A39 G23 C26 T40 | A45 G22 C23 T22 | 8 |
| Camelpox virus | CMS | A39 G24 C26 T39 | A45 G22 C23 T22 | 9 |
| Cowpox virus | Brighton Red | A43 G22 C26 T37 | A40 G25 C18 T29 | 10 |
| Cowpox virus | Germany 91-3 | A40 G24 C27 T37 | A43 G23 C24 T22 | 11 |
| Cowpox virus | GRI-90 | A40 G23 C27 T38 | A44 G22 C24 T22 | 12 |
| Ectromelia virus | Moscow | A40 G23 C27 T38 | A41 G24 C17 T30 | 13 |
| Horsepox virus | MNR-76 | A40 G24 C26 T38 | A45 G21 C24 T22 | 14 |
| Rabbitpox virus | Utrecht | A40 G24 C26 T38 | A43 G23 C24 T22 | 15 |
| Raccoonpox virus | Herman | A45 G23 C22 T38 | A43 G24 C18 T27 | 16 |
| Taterapox virus | Dahomey 1968 | A39 G24 C26 T39 | A44 G22 C24 T22 | 17 |
| Vaccinia virus | Copenhagen | A39 G25 C27 T37 | A45 G21 C24 T22 | 18 |
