# Supplementary material for: Comprehensive Biothreat Cluster Identification by PCR/Electrospray-Ionization Mass Spectrometry
Source: PLoS One. 2012 Jun 29;7(6):e36528. doi: 10.1371/journal.pone.0036528 (PMC3387173; doi:10.1371/journal.pone.0036528)
Supplement: Table S18 — Expected Influenza A virus (H5N1) signatures and potential overlap with non-H5N1 species. (DOCX) [file pone.0036528.s022.docx]

Table S18. Expected Influenza A virus (H5N1) signatures and potential overlap with non-H5N1 species

| **Organism** | **Number of Strains^1^** | **FLU_ALL_PB1 (VIR2798)** | **FLU_A_NP (VIR1266)** | **Overlap with Non-H5N1 Species** |
| --- | --- | --- | --- | --- |
| Influenza A virus (H5N1) | 267 | A37 G32 C26 T33 | A34 G23 C21 T23 | None |
|  | 246 | A38 G31 C26 T33 | A34 G24 C21 T22 | 20 strains- 15 Avian H9N2 (2003; Shantou); 5 Swine H1N1 (Virginia 1987; England 1992-95) |
|  | 240 | A38 G31 C27 T32 | A34 G24 C21 T22 | None |
|  | 147 | A38 G31 C27 T32 | A35 G23 C21 T22 | 9 Avian H9N2 (2006; Guangxi) |
|  | 103 | A38 G31 C25 T34 | A34 G24 C21 T22 | None |
|  | 41 | A38 G31 C27 T32 | A34 G24 C20 T23 | None |

**^1^** Based on analysis of GenBank sequence data.
