# Supplementary material for: Comprehensive Biothreat Cluster Identification by PCR/Electrospray-Ionization Mass Spectrometry
Source: PLoS One. 2012 Jun 29;7(6):e36528. doi: 10.1371/journal.pone.0036528 (PMC3387173; doi:10.1371/journal.pone.0036528)
Supplement: Table S19 — Expected Filovirus signatures. (DOCX) [file pone.0036528.s023.docx]

Table S19. Expected Filovirus signatures

| **Organism** | **Strain** | **Filovirus (VIR853)** | **Filovirus (VIR858)** |
| --- | --- | --- | --- |
| Zaire ebolavirus | Gabon 2001 | A19 G15 C15 T26 I4 | A31 G25 C22 T25 |
|  | Gabon 1995 | A20 G15 C14 T26 I4 | A31 G25 C21 T26 |
|  | Gabon 2003 | A20 G15 C14 T26 I4 | A31 G25 C22 T25 |
|  | Mayinga^1^;Zaire 1995 | A20 G15 C14 T26 I4 | A32 G24 C22 T25 |
| Reston ebolavirus | Pennsylvania; Reston^1^ | A18 G16 C13 T28 I4 | A30 G25 C22 T26 |
| Sudan ebolavirus | Boniface^1^; Maleo; Gulu; Gulu 10/2000; Yambio | A18 G15 C12 T27 I4 | Does Not Amplify |
| Cote d'Ivoire ebolavirus | Ivory Coast | A20 G15 C15 T25 I4 | Does Not Amplify |
| Bundibugyo ebolavirus |  | A20 G16 C13 T26 I4 | Does Not Amplify |
| Lake Victoria marburgvirus | Ravn^1^ | A19 G14 C10 T29 I4 | A34 G24 C20 T25 |
|  | Voege | A19 G14 C10 T29 I4 | A34 G24 C22 T23 |
|  | Musoke; pp3 guinea pig lethal variant; pp4 guinea pig nonlethal variant | A19 G14 C12 T30 I4 | A32 G26 C22 T23 |
|  | 05DRC99;07DRC99;M/S. Africa/Johannesburg/1975/Ozolin | A19 G14 C13 T29 I4 | A32 G26 C23 T22 |
|  | Ang0126; Ang0214; Ang0215; Ang0754; Ang0998;Ang1379c; Ang1381; Ang1386 | A19 G14 C13 T29 I4 | A33 G25 C23 T22 |
|  | 09DRC99; R1; R2; R3; Ravn | A19 G14 C13 T29 I4 | A34 G24 C20 T25 |
|  | Ci67; POPP | A19 G14 C13 T29 I4 | A34 G24 C22 T23 |

^1^ Samples obtained from CDC and tested experimentally.
